# Multiple Independent Retroelement Insertions in the Promoter of a Stress Response Gene Have Variable Molecular and Functional Effects in *Drosophila*

**DOI:** 10.1371/journal.pgen.1006249

**Published:** 2016-08-12

**Authors:** Miriam Merenciano, Anna Ullastres, M. A. R. de Cara, Maite G. Barrón, Josefa González

**Affiliations:** 1 Institute of Evolutionary Biology (CSIC-Universitat Pompeu Fabra), Barcelona. Spain; 2 Laboratoire d’Eco-anthropologie et Ethnobiologie, UMR 7206, CNRS/MNHN/Universite Paris 7, Museum National d’Histoire Naturelle, F-75116 Paris, France; University of Utah School of Medicine, UNITED STATES

## Abstract

Promoters are structurally and functionally diverse gene regulatory regions. The presence or absence of sequence motifs and the spacing between the motifs defines the properties of promoters. Recent alternative promoter usage analyses in *Drosophila melanogaster* revealed that transposable elements significantly contribute to promote diversity. In this work, we analyzed in detail one of the transposable element insertions, named *FBti0019985*, that has been co-opted to drive expression of *CG18446*, a candidate stress response gene. We analyzed strains from different natural populations and we found that besides *FBti0019985*, there are another eight independent transposable elements inserted in the proximal promoter region of *CG18446*. All nine insertions are solo-LTRs that belong to the *roo* family. We analyzed the sequence of the nine *roo* insertions and we investigated whether the different insertions were functionally equivalent by performing 5’-RACE, gene expression, and cold-stress survival experiments. We found that different insertions have different molecular and functional consequences. The exact position where the transposable elements are inserted matters, as they all showed highly conserved sequences but only two of the analyzed insertions provided alternative transcription start sites, and only the *FBti0019985* insertion consistently affects *CG18446* expression. The phenotypic consequences of the different insertions also vary: only *FBti0019985* was associated with cold-stress tolerance. Interestingly, the only previous report of transposable elements inserting repeatedly and independently in a promoter region in *D*. *melanogaster*, were also located upstream of a stress response gene. Our results suggest that functional validation of individual structural variants is needed to resolve the complexity of insertion clusters.

## Author Summary

The presence of several transposable element insertions in the promoter region of a *Drosophila melanogaster* gene has only been described in *heat shock protein* genes. In this work, we have discovered and characterized in detail several naturally occurring independent transposable element insertions in the promoter region of a cold-stress response gene in the fruitfly *Drosophila melanogaster*. The nine transposable element insertions described are clustered in a small 368 bp region and all belong to the same family of transposable elements: the *roo* family. Each individual insertion is present at relatively low population frequencies, ranging from 1% to 17%. However, the majority of strains analyzed contain one of these nine *roo* insertions suggesting that this region might be evolving under positive selection. Although the sequence of these insertions is highly similar, their molecular and functional consequences are different. Only one of them, *FBti0019985*, is associated with increased viability in nonstress and in cold-stress conditions.

## Introduction

Promoters are crucial regions for the transcriptional regulation of gene expression. Recent computational and experimental advances in functional genomics techniques have allowed defining the promoter architecture to an unprecedented level. Several core promoter motifs such as the *Initiator* (*Inr*) and the *Downstream core Promoter Element* (*DPE*) have been described, and it is likely that many others remain to be discovered. The presence or absence of the core promoter motifs influences enhancer-promoter communication and thus gene regulation [1]. Promoter regions also harbour transcription factor binding motifs, which are another important component in the regulation of gene expression [2]. Besides cis-regulatory elements that influence the temporal and spatial expression patterns of genes, proximal promoters often contain alternative transcription start sites (TSSs) [1, 3]. Rather than being “biological noise” from imprecise binding of the transcription initiation machinery, genome-wide analyses of TSSs usage showed that alternative TSSs play an important role in the diversification of gene expression patterns [4–8].

Transposable elements (TEs), long proposed to play an important role in gene regulation [9, 10], have recently been found to provide at least 1,300 alternative TSSs in the *Drosophila melanogaster* genome [8]. TEs can also add Transcription Factor Binding Sites (TFBSs) to the promoter of genes as has been recently shown in Drosophila and humans [11–13]. As a result of adding particular sequence elements, many TEs confer their intrinsic regulatory properties to nearby genes demonstrating that they distribute cis-regulatory modules [8]. Finally, TEs inserted in promoter regions can also influence gene expression by disrupting the promoter architecture. This is the case, for example, of naturally occurring *P-element* insertions in the promoter of *heat shock protein* (*hsp*) genes [14].

**Fig 1:**
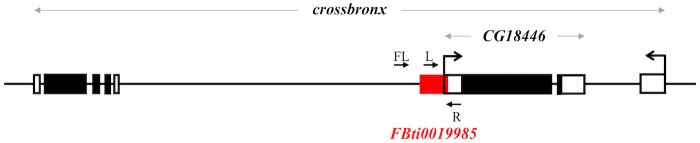
*FBti0019985* is inserted in the first intron of *cbx* gene and it overlaps with *CG18446* 5’-UTR region. Schematic representation of the genomic region where *FBti0019985* is inserted: chromosome 2R: 9,864,510–9,875,072. *FBti0019985* is shown in red. Black boxes represent exons and white boxes represent the 5’-UTRs and the 3’-UTRs. Primers used to check for the presence/absence of *FBti0019985* are depicted as black arrows (FL, R, and L; see Materials and Methods).

One of the TEs identified as providing an alternative TSS by Batut *et al* (2013) [8], named *FBti0019985*, was previously reported in a screening designed to identify putatively adaptive TE insertions in *D*. *melanogaster* [15]. However, this particular TE was not further studied because its population frequency could not be accurately determined [15]. *FBti0019985* is a *roo* solo-LTR inserted in the 5’-UTR of *CG18446* gene, which is nested in the first intron of *crossbronx* (*cbx*) (Fig 1). TEs from the *roo* family have long been proposed to affect the expression of nearby genes by adding and distributing cis-regulatory regions [16–19]. Specifically, *roo* LTRs contain several TFBSs and the *Inr* sequence characteristic of core promoters [8, 20].

Interestingly, *CG18446* has been identified as a candidate gene for cold resistance: it is upregulated in fly strains that have been selected for increased cold resistance compared with control strains that were not subjected to cold-stress [21]. Cold resistance is an ecologically and evolutionarily relevant trait because it influences the ability of the species to adapt to different climatic conditions and thus, their geographical distribution [22, 23]. There is good evidence suggesting that *D*. *melanogaster* adapts to cold environments and a growing list of candidate genes involved in this thermotolerance phenotype is being identified [21, 24–28]. However, the molecular variants responsible for the adaptive cold-stress resistance phenotype remain elusive [29].

In this work, we further analyzed the presence/absence of *FBti0019985* in four natural populations of *D*. *melanogaster*. We found that besides *FBti0019985*, eight other *roo* elements have inserted in a 368 bp region around *CG18446* transcript start site. These *roo* elements differ in the insertion site and in their orientation. On the other hand, all elements have the same size and show high sequence conservation: all cis-regulatory elements previously described in *roo* LTRs are highly conserved [8, 30]. We further investigated whether these different insertions were functionally equivalent by performing 5’-RACE, gene expression, and phenotypic analyses. Our results showed that the functional consequences of the different *roo* insertions depend on the particular position where the element is inserted. Among the nine different *roo* solo-LTR insertions, only *FBti0019985* is consistently associated with increased viability in nonstress and cold-stress conditions across genetic backgrounds.

## Results

### Besides *FBti0019985*, eight other *roo* solo-LTRs are inserted in the promoter region of *CG18446*

We first aimed at estimating the frequency of *FBti0019985* in non-African natural *D*. *melanogaster* populations. Thus, we checked using PCR whether this insertion was present, polymorphic, or absent in 28 strains from a natural population collected in North Carolina (North America, DGRP strains [31, 32]) and in 15 strains from a natural population collected in Bari (Italy, Europe [33]) (Table 1). We obtained PCR results for 39 of the 43 strains tested: nine strains produced PCR bands consistent with *FBti0019985* being present, five strains appeared as heterozygous, 13 strains showed unexpected band patterns, and 12 strains appeared as absent (Table 1) (see Material and Methods). To verify these results, we sequenced 32 of the 39 strains including all the strains that showed some evidence of presence (Table 1).

**Table 1:** The nine *roo* solo-LTR insertions analyzed in this work.

Insertion	Fly strain	PCR results	Sequenced band	Insertion position^a^
***FBti0019985***	*RAL-639*	Present	FL-R	Reference position
	*RAL-802*	Present	FL-R / L-R	Reference position
	*RAL-810*	Present	FL-R	Reference position
	*IV68*	Present	FL-R / L-R	Reference position
***roo***_***+7***_	*RAL-405*	Present	FL-R	+ 7 bp
	*RAL-887*	Present	FL-R	+ 7 bp
	*RAL-911*	Present	FL-R	+7 bp
	*RAL-441*^b^	Larger L-R	FL-R	+ 7 bp
		Larger FL-R		
	*RAL-801*^b^	Larger L-R	FL-R	+ 7 bp
		Larger FL-R		
***roo***_***+175***_	*IV145*	Heterozygous	Larger FL-R	+ 175 bp
***roo***_***+278***_	*RAL-502*	Smaller L-R	L-R	+ 278 bp
		No FL-R		
***roo***_***-19***_	*IV42*	Present	FL-R	- 19 bp inverted
	*IV127*	Present	FL-R	- 19 bp inverted
***roo***_***-28***_	*IV40*	Heterozygous	Larger FL-R	- 28 bp inverted
***roo***_***-44***_	*RAL-195*	Only FL-R	FL-R	- 44 bp inverted
	*RAL-383*	Only FL-R	FL-R	- 44 bp inverted
***roo***_***-68***_	*RAL-75*	Only FL-R	FL-R	- 68 bp inverted
	*RAL-716*	Only FL-R	FL-R	- 68 bp inverted
	*IV69*	Heterozygous	Larger FL-R	- 68 bp inverted
***roo***_***-90***_	*RAL-21*	Larger L-R	FL-R	- 90 bp
	*RAL-88*	Larger L-R	FL-R	- 90 bp
	*RAL-177*	Larger L-R	FL-R	- 90 bp
	*RAL-737*	Larger L-R	FL-R	- 90 bp
	*RAL-820*	Larger L-R	FL-R / L-R	- 90 bp
	*RAL-857*	Heterozygous	FL-R / L-R	- 90 bp
	*IV50*	Heterozygous	Larger FL-R	- 90 bp
**Absent**	*RAL-40*	Smaller L-R	FL-R / L-R	Absent
	*RAL-371*	Absent	FL-R	Absent
	*RAL-391*	Absent	FL-R	Absent
	*RAL-508*	Absent	NS	Absent
	*RAL-783*	Absent	FL-R	Absent
	*RAL-822*	Absent	NS	Absent
	*RAL-855*	Absent	NS	Absent
	*RAL-908*	Absent	FL-R	Absent
	*IV22*	Absent	FL-R	Absent
	*IV49*	Absent	NS	Absent
	*IV52*	Absent	NS	Absent
	*IV72*	Absent	NS	Absent
	*IV75*	Absent	NS	Absent
**No data**	*RAL-776*	No results		
	*IV33*	No results		
	*IV125*	No results		
	*IV148*	No results		

NS, not sequenced

^a^ "+" indicates the insertion is downstream of *FBti0019985* and "-" indicates the insertion is upstream of *FBti0019985*

^b^These strains have a 95 bp duplication upstream of the insertion

**Fig 2:**
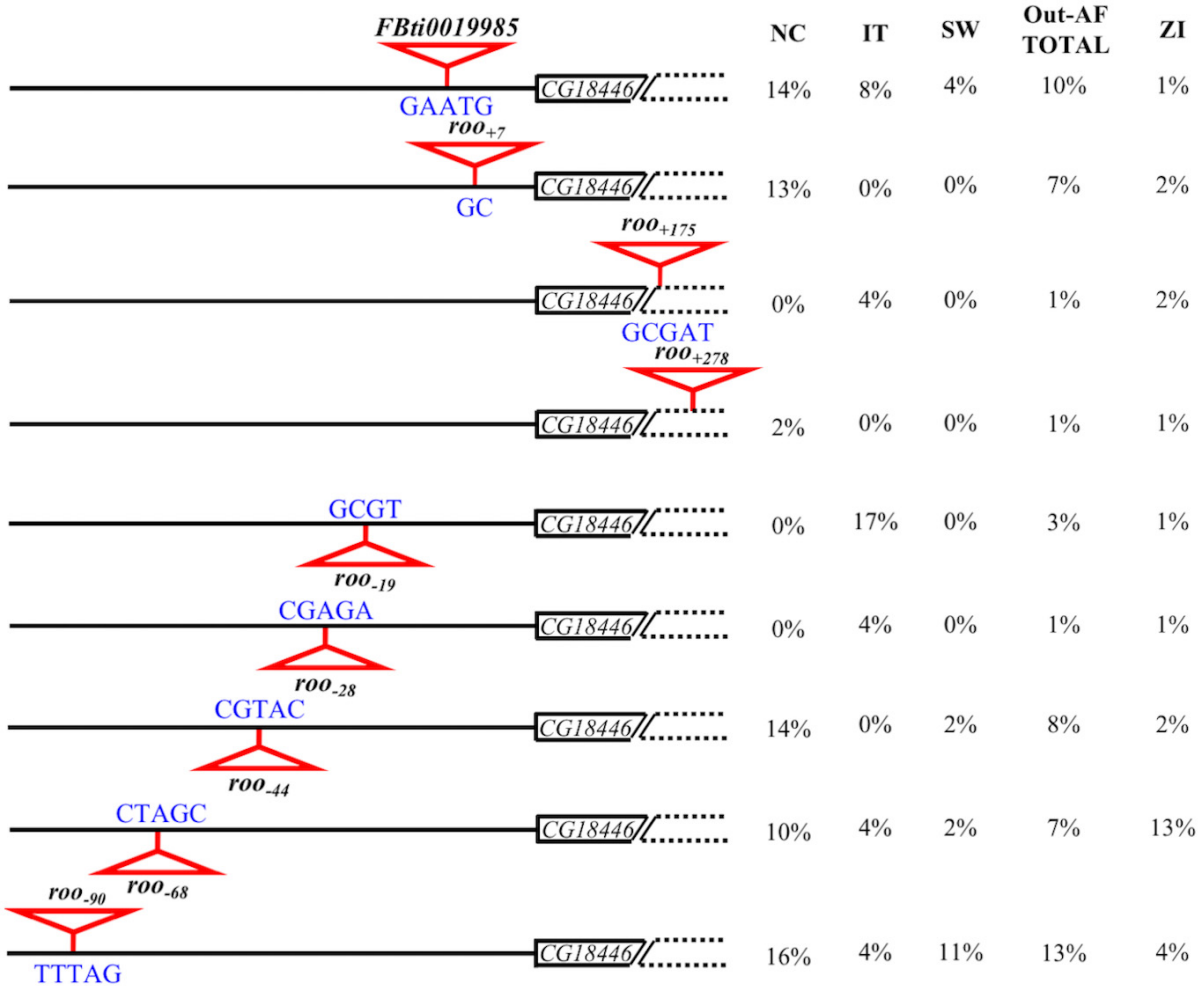
Besides *FBti0019985*, eight other *roo* solo-LTR are inserted in the proximal promoter of *CG18446*. Schematic representation of the genomic region where the nine *roo* solo-LTRs are inserted. *roo* insertions are depicted as red triangles. White boxes represent *CG18446* 5’-UTR. Regions depicted with dotted lines are not drawn to scale. Target Site Duplications (TSDs) are shown in blue. NC, allele frequency (%) in the North American population; IT, allele frequency (%) in the Italian population; SW, allele frequency (%) in the Swedish population; Out-AF total, allele frequency (%) in all the out-of-Africa populations; ZI, allele frequency (%) in the Zambia population.

Only four of the nine strains classified as present, according to the PCR results, had the *FBti0019985* insertion. For the rest of this work, we considered the position where *FBti0019985* is inserted as the "reference position". The other five present strains, the five heterozygous strains, and 12 of the 13 strains that gave unexpected PCR bands contained different *roo* solo-LTR insertions (Table 1). Overall, besides *FBti0019985*, we found eight other 428 bp *roo* solo-LTRs inserted in eight different positions (Fig 2). Three *roo* insertions are located downstream of the reference position: *roo*_*+7*_, *roo*_*+175*_, and *roo*_*+278*_ (Fig 2). Two of the four strains carrying *roo*_*+7*_ have a duplication of the 95 bp region located immediately upstream of the insertion (Table 1). *roo*_*+175*_ element is inserted in the 5’-UTR region, and *roo*_*+278*_ is inserted in the first exon of *CG18446* gene. Both *roo*_*+175*_ and *roo*_*+278*_ have a conserved *Inr* motif. If transcription starts in these insertions, flies carrying *roo*_*+175*_ would have a 100 bp shorter 5'-UTR, and flies carrying *roo*_*+278*_ would have a 35 amino acids shorter *CG18446* protein. The other five *roo* insertions are located upstream of the reference position: *roo*_*-19*_, *roo*_-*28*_, *roo*_-44_, *roo*_*-68*_, *roo*_*-90*_ (Fig 2). Four of them, *roo*_-19_, *roo*_-28_, *roo*_*-44*_, and *roo*_-*68*_, are inserted in reverse orientation.

We used *Tlex-2* software to further analyze the frequency of the nine *roo* insertions in 21 additional DGRP strains, in 26 strains from a Swedish natural population, and in 42 strains from a population collected in the ancestral range of the species, Zambia (Fig 2 and S1 Table) (see Material and Methods) [34]. Overall, we found that 67 strains, out of the 128 strains analyzed, contained one of the nine *roo* solo-LTR insertions. The two most common *roo* insertion in out-of-Africa populations are *roo*_-90_ and *FBti0019985* present in 13% and 10% of the strains tested, respectively (Fig 2). Besides, some insertions are only present in the North Carolina natural population while others are specific to the Italian natural population (Fig 2). Only three of the nine insertions described in North Carolina and Italian populations are present in the Swedish population. However, we did not perform *de novo* discovery of TEs in this population. Thus, it could be that other private insertions are present in the Swedish population. Finally, all the nine insertions were present in the African population although most of them were present at very low frequencies (Fig 2).

In summary, we have found that besides the *FBti0019985* insertion annotated in the reference genome, eight other 428 bp *roo* solo-LTRs are inserted nearby *CG18446* TSS in natural populations of *D*. *melanogaster* (Fig 2) [35]. Each one of the strains analyzed contains a single solo-LTR *roo* insertion and most of the analyzed strains contain one of the nine solo-LTR *roo* insertions.

### The nine *roo* solo-LTR are independent insertions that occurred at different evolutionary timepoints

We identified the Target Site Duplications (TSD) of the nine different *roo* insertions using data from the 26 present strains sequenced in this work (Table 1). We could identify the TSD for all *roo* insertions except for *roo*_*+278*_. We found that six of the eight TSDs identified are five nucleotides long as has been previously described for this family [36] (Fig 2). However, the TSD sequences did not match the proposed TSD consensus sequence [34, 36, 37]. We thus used all the available *roo* TSD sequences to build a new consensus (S1 Fig). The different *roo* solo-LTR insertions had different TSDs suggesting that they are independent insertions (Fig 2). Furthermore, all the *roo* elements located in a given insertion site have the same exact TSD and are inserted in the same orientation suggesting that each one of them is a unique insertion event (Fig 2).

To test whether these nine insertion events were the result of a burst of transposition, we constructed a phylogenetic tree. We included the nine *roo* insertions sequenced in this work and 115 other *roo* insertions present in the *D*. *melanogaster* genome (S2 Fig and S1 Text). We found that not all the newly described *roo* insertions clustered together suggesting that they did not insert at the same time (S2 Fig and S1 Text).

All the TEs identified in *CG18446* proximal promoter region belong to the *roo* family. Thus, we also investigated whether *roo* elements annotated in the reference genome are preferentially inserted into gene proximal promoter regions as has been previously described for other TE families [38, 39]. We analyzed the 138 insertions belonging to the *roo* family annotated in the *D*. *melanogaster* reference genome (v5). We found 21 *roo* insertions located in the 1 kb region upstream of a gene or overlapping the 5’-end of a gene. Thus, only 15.2% of the *roo* elements in the *D*. *melanogaster* genome are located in gene promoters and/or 5’-UTRs.

In summary, TSD analyses of the nine insertions characterized in this work suggested that they are independent insertions, and confirmed the length but not the sequence previously reported as the TSD consensus for this family. Our results are not consistent with the nine *roo* insertions being the result of a single burst of transposition. Finally, our analyses also suggested that *roo* elements do not preferentially insert in 5’ gene regions.

### The nine *roo* insertions add the same cis-regulatory sequences

**Fig 3:**
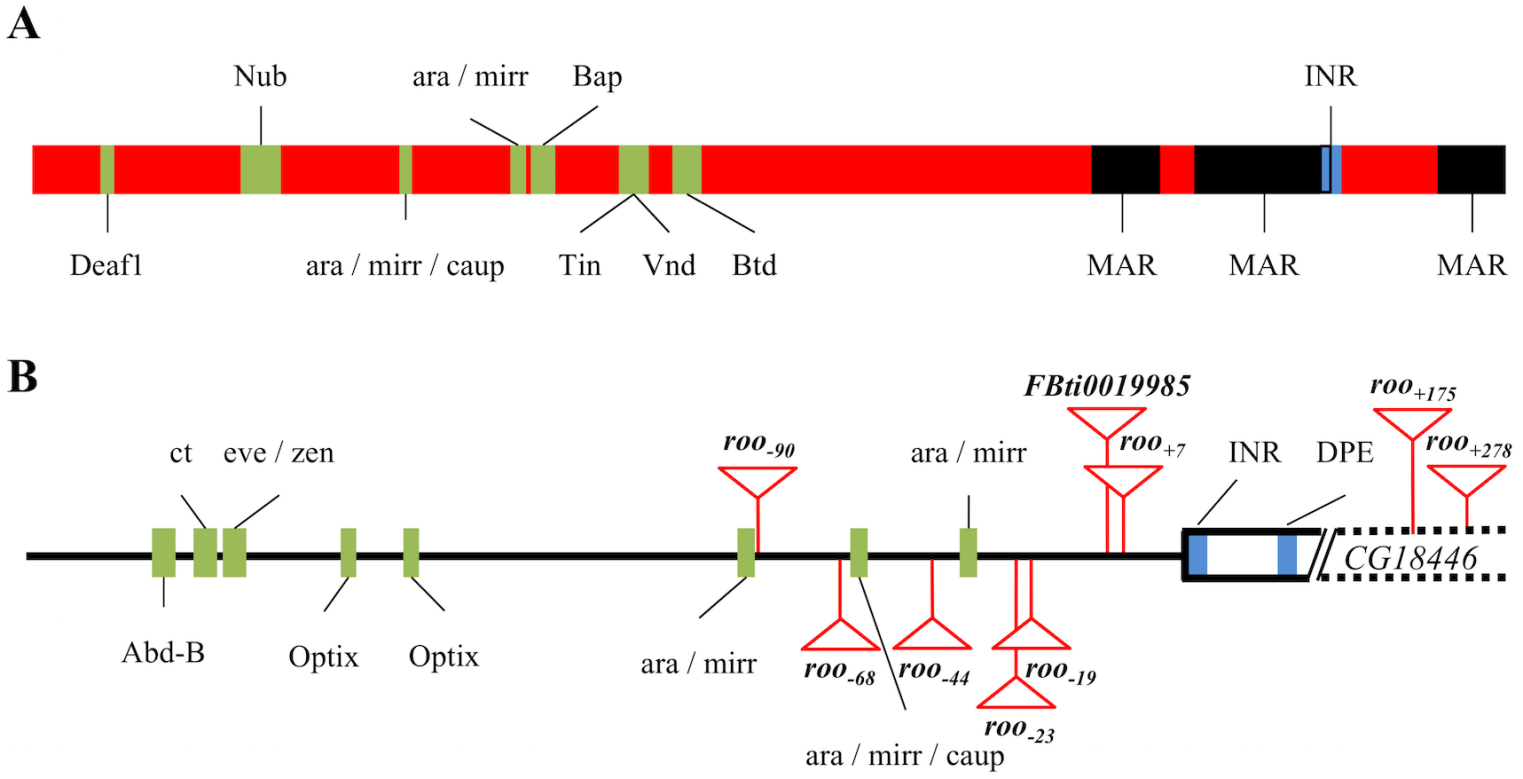
Conserved regulatory regions in the the nine *roo* solo-LTR insertions and in the proximal promoter region of *CG18446*. (A) Location of the nine transcription factor binding sites (green boxes), the *Inr* motif (blue box), and regions with matrix association potential (MARs) (black boxes), in the *roo* solo-LTR consensus sequence. *Deaf1*, *ara*, *mirr* and *caup* TFBS have been identified in this work. (B) Location of the eight transcription factor binding sites (green boxes) and the two core promoter motifs (blue boxes) in the proximal promoter region of *CG18446*. Different *roo* insertions are depicted as red triangles. The positions of *roo*_*+175*_ and *roo*_*+278*_ are not drawn to scale.

We analyzed multiple sequence alignments of all the *roo* insertions located nearby *CG18446*. We identified TFBSs using the JASPAR database (see Material and Methods). We also specifically looked for conservation of the regulatory regions previously described in the *roo* family [8, 30], and for conserved core promoter motifs [1] (Fig 3A and S2A Table). Overall, there was very little diversity among the nine solo-LTRs (S3A Fig). The five TFBSs and the *Inr* sequence previously identified in the consensus sequence of *roo* LTRs are conserved in all the *roo* copies located in the proximal promoter of *CG18446* [8]. Additionally, we found another four TFBSs that are also highly conserved in all the copies (Fig 3A and S3A Fig). The nine transcription factors are involved in developmental processes. Additionally, *Deaf1* and *Nub* are also involved in immune response [40, 41]. Finally, three previously identified Matrix Associated Regions (MARs) in LTRs from the *roo* family are also highly conserved in the nine insertions (Fig 3A and S3B Fig) [30]. These results suggest that these *roo* solo-LTR insertions are introducing the same cis-regulatory regions in the *CG18446* proximal promoter region. Still, the functional effect of these insertions might be different because they are located in different positions and have different orientations (Fig 2).

### *roo* insertions affect the spacing of Transcription Factor Binding Sites in the proximal promoter region of *CG18446*

We analyzed the proximal promoter region of *CG18446* in the 30 strains sequenced in this work. We could not identify the TATA box suggesting that *CG18446* has a DPE promoter [1]. We identified eight TFBSs in the proximal promoter of *CG18446* (Fig 3B and S2C Table). These eight TFBSs are highly conserved in all the strains analyzed (S3C Fig). The different *roo* insertions characterized in this work do not disrupt any of the identified core promoter motifs or TFBSs (Fig 3B). However, they do affect the spacing between the different regulatory motifs, which might affect the protein-protein interaction at the *CG18446* promoter and thus the expression level of this gene (Fig 3B) [14].

### *roo* insertions could be recruiting the HP1a protein

**Fig 4:**
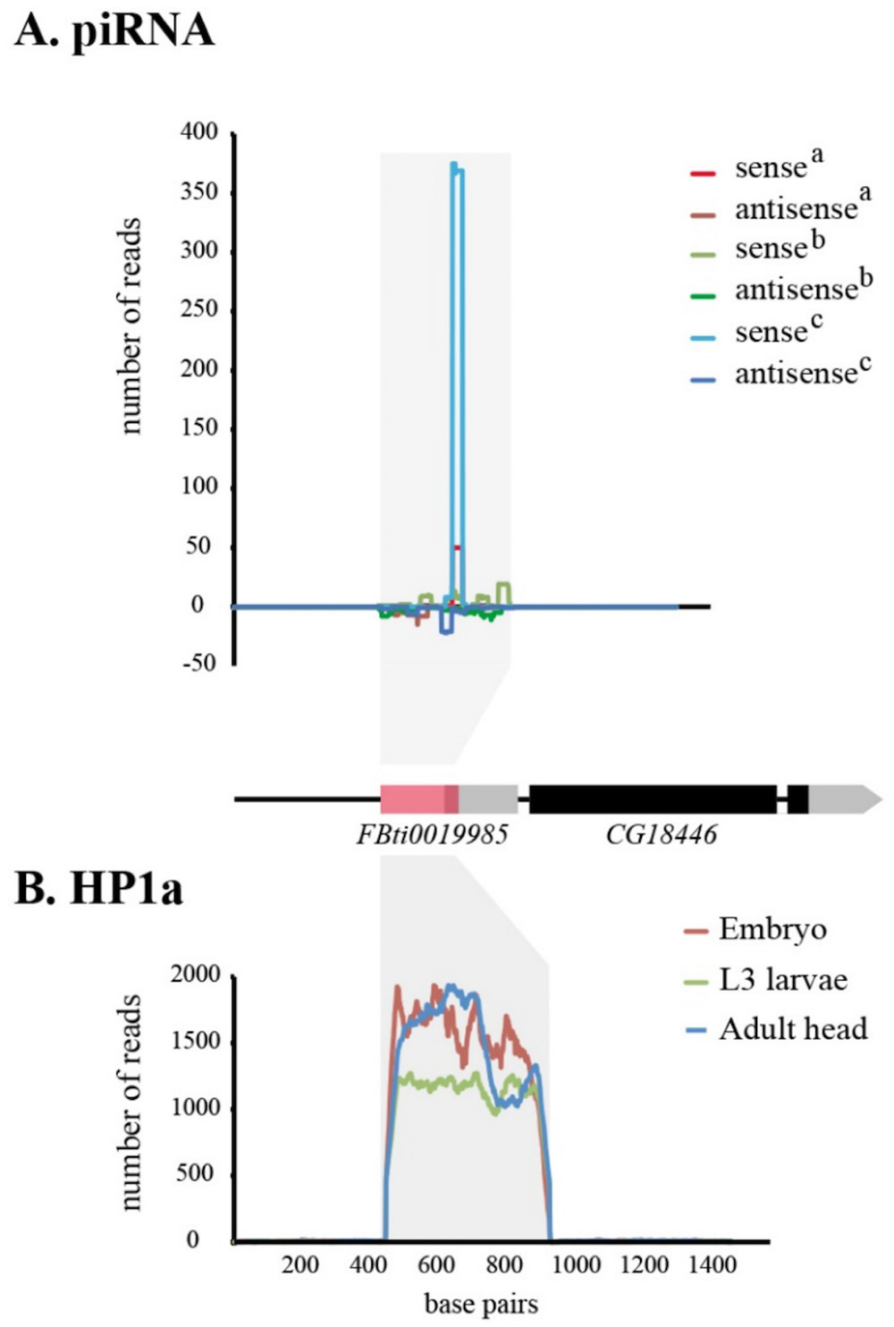
Mapping of piRNA reads and HP1a reads to the *FBti0019985* region. (A) Number of piRNA reads mapped to a 1.4 kb region including *FBti0019985*. ^*a*^Li *et al* (2009) piRNA library, ^b^Satyaki *et al* (2014) piRNA library and ^c^Shpiz *et al* (2014) piRNA library. (B) Number of HP1a reads mapped to the same 1.4 kb region.

Besides affecting the spacing of transcription factor binding site, another mechanism by which *roo* insertions could be affecting *CG18446* expression is by recruiting piRNAs that would lead to heterochromatin formation [42, 43]. We mapped piRNA reads from three different available libraries to a 1.4 kb region including *FBti0019985* (Fig 4A) (see Material and Methods) [44–46]. We found that most of the piRNAs mapping to the insertion were sense reads, suggesting that *FBti0019985* is not acting as a target for heterochromatin assembly [42].

We also looked for evidence of HP1a binding to *FBti0019985* using modENCODE data (see Material and Methods) [47]. HP1a is a structural chromosomal protein that mediates both gene expression and gene silencing [48]. We did find evidence of HP1a reads binding to *FBti0019985* (Fig 4B). Thus, by recruiting HP1a, *FBti0019985* could be affecting the expression of *CG18446*. The same results were obtained for the other eight *roo* solo-LTR insertions: most of the piRNAs mapping to the insertions were sense reads and we found evidence of HP1a binding to all of them (S3 Table). Overall, our results are suggestive but not conclusive of HP1a binding to the nine *roo* insertions described in this work.

To further investigate the possible functional consequences of the *roo* insertions, we focused on the five insertions present at higher population frequencies in out-of-Africa populations: *FBti0019985*, *roo*_+7_, *roo*_-44_, *roo*_-90_, and *roo*_-68_ (Fig 2).

### Only *FBti0019985* and *roo*_*+7*_ affect the transcription start site of *CG18446*

**Fig 5:**
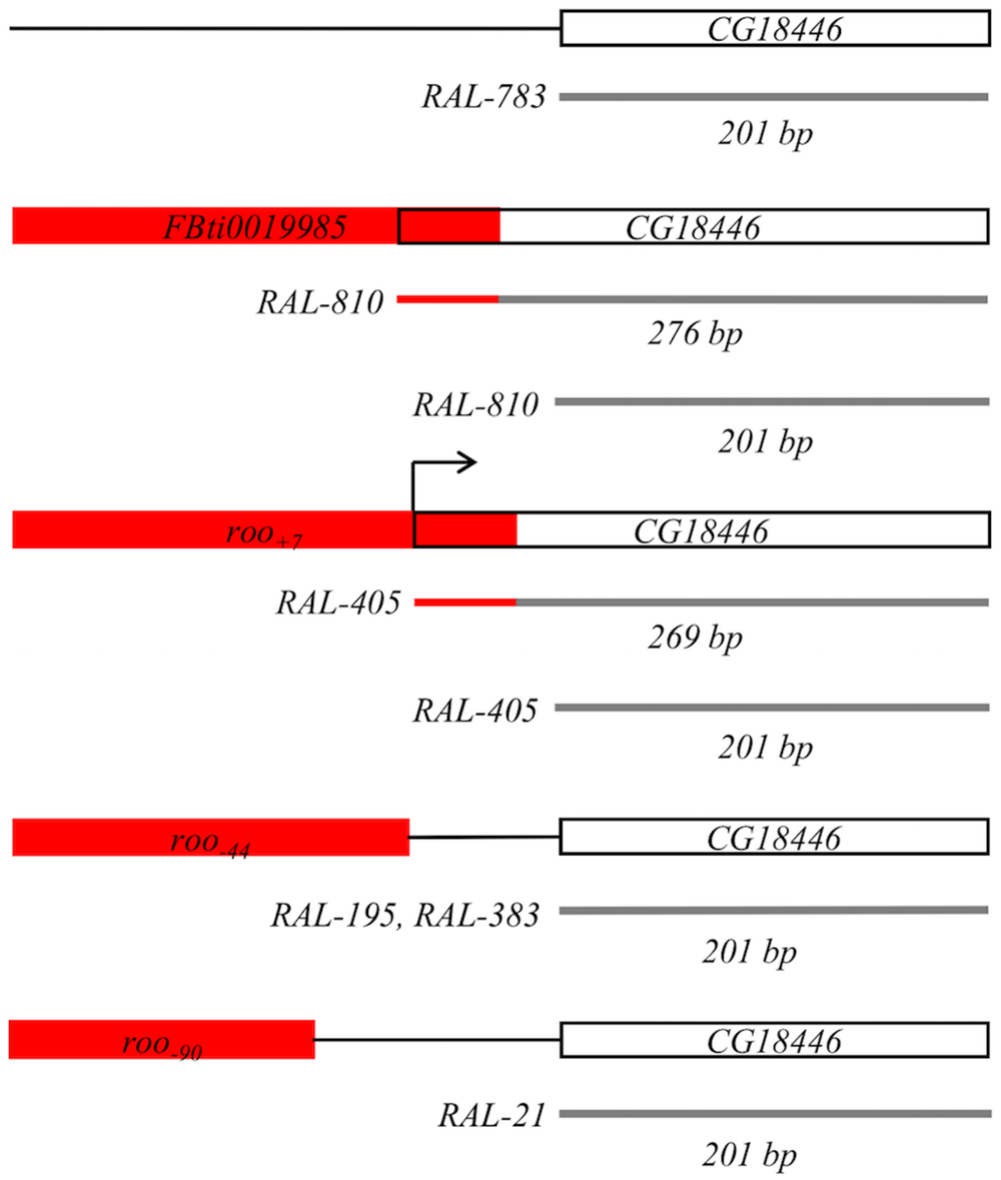
*FBti0019985* and *roo*_*+7*_ affect the transcription start site of *CG18446*. Schematic representation of the results obtained using the 5’-RACE technique. Red boxes represent different *roo* insertions and white boxes represent *CG18446* 5’-UTRs. Partial transcripts obtained by 5’-RACE are depicted as grey lines. The region of the transcript that overlaps with a *roo* insertion is shown as a red line. The last 50 bp of *FBti0019985* and *roo*_*+7*_ are included in the 5’-UTR of *CG18446*.

We investigated whether *roo* insertions could be providing an alternative TSS to *CG18446*. Batut *et al* (2013) [8] reported that the TSS of *CG18446* is located inside *FBti0019985*. However, this finding was obtained using RAMPAGE and was not further validated using 5’-RACE. For this reason, we performed a 5’-RACE with the *RAL-810* strain that carries *FBti0019985* and with the *RAL-783* strain that does not carry any of the nine *roo* solo-LTR insertions. As expected, we found that the TSS of *CG18446* is inside the TE: the first 50 bp of the 276 bp 5’-UTR correspond to *FBti0019985* (Fig 5). Additionally, flies with the insertion have also a shorter transcript, with a 201 bp 5’-UTR, that does not start in *FBti0019985* (Fig 5). Most of the sequenced transcripts start in the *FBti0019985* insertion (14 out of 20 transcripts analyzed). Flies without the *FBti0019985* insertion only have the 201 bp 5’-UTR transcript (Fig 5).

We then checked whether *roo*_*+7*_, located only 7 bp downstream of *FBti0019985*, *roo*_*-90*_, which is the most distal insertion, and *roo*_*-44*_, which is inserted in reversed orientation, also provide an alternative TSS to *CG18446*. We found that *roo*_*+7*_ affects the TSS of *CG18446* (Fig 5). Indeed, the TSS in *roo*_*+7*_ is in the same nucleotide position as in *FBti0019985*. Thus, *CG18446* transcript in flies with *roo*_*+7*_ is 7 bp shorter compared with the transcript in flies with *FBti0019985*. Similarly to *FBti0019985*, most of the sequenced transcripts started in the *roo*_*+7*_ insertion (18 out of 22 transcripts analyzed). On the other hand, we did not find evidence of a TSS inside *roo*_*-90*_, which might indicate that the distance of the TE to the nearby gene affects its ability to provide an alternative TSS (Fig 5). Finally, we analyzed two different strains carrying the *roo*_*-44*_ insertion in the same position and we could not find evidence for a transcript with the TSS in *roo*_*-44*_ (Fig 5).

Overall, we found that only *FBti0019985* and *roo*_*+7*_ insertions modify the length of *CG18446* transcript. These two *roo* insertions are located a few nucleotides from the gene and both are inserted in 5’ to 3’ orientation.

### *FBti0019985* is associated with changes in embryonic *CG18446* expression

**Fig 6:**
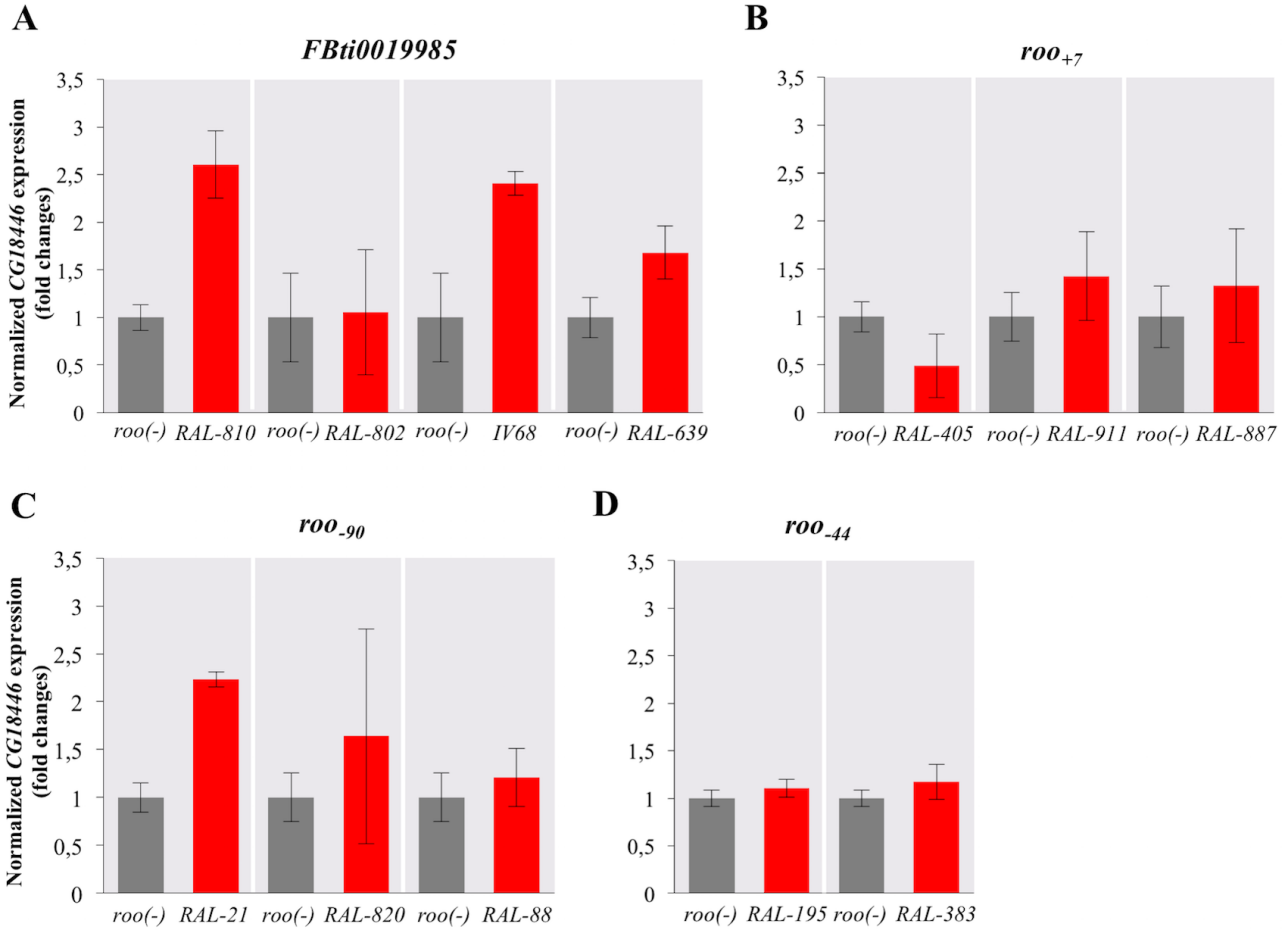
*FBti0019985* is associated with changes in *CG18446* expression. Normalized *CG18446* expression level relative to *Act5C* in embryos without *roo* insertion (grey) and in embryos with different *roo* insertions (red). (A) For *FBti0019985*, we compared the expression of *CG18446* in flies with four different genetic backgrounds. In three backgrounds, the presence of *FBti0019985* was associated with *CG18446* upregulation. These results were significant in two backgrounds, *RAL-810* and *IV68*, and marginally significant in the third background, *RAL-639*. (B) *roo*_*+7*_ was only associated with changes of expression in one of the three backgrounds analyzed: *RAL-405*. (C) *roo*_*-90*_ was also only associated with changes of expression in one of the three backgrounds analyzed: *RAL-21*. (D) Finally, *roo*_*-44*_ was not associated with changes in expression in any of the two backgrounds analyzed. Error bars represent the standard error of the mean (SEM) for the three biological replicates performed for each experiment.

We further analyzed whether different *roo* insertions were associated with changes in *CG18446* expression in embryos, where this gene is highly expressed [49]. For *FBti0019985*, we analyzed the expression of *CG18446* in flies with four different genetic backgrounds. In three of the four backgrounds, *FBti0019985* is associated with upregulation of *CG18446* (Fig 6A). This result is significant in two genetic backgrounds, *RAL-810* and *IV68*, and marginally significant in a third background, *RAL-639* (t-test p-value = 0.045, p-value = 0.005 and p-value = 0.062, respectively) (Fig 6A). On the other hand, only in one of the three genetic backgrounds analyzed for *roo*_*+7*,_ the insertion is associated with downregulation of this gene (t-test p-value = 0.015 for *RAL-405*) (Fig 6B).

We also checked the expression of *CG18446* in flies with two *roo* solo-LTR insertions that do not provide an alternative TSS to this gene: *roo*_*-90*_ and *roo*_*-44*_. We found that *roo*_*-90*_ is only associated with *CG18446* upregulation in one of the three backgrounds analyzed (p-value = 0.001, for *RAL-21*) (Fig 6C). Two different strains with the *roo*_*-44*_ solo-LTR insertion did not show differences in the level of expression of *CG18446* compared with strains without the insertion (p-values > 0.05 in both cases) (Fig 6D).

Overall, we found that *FBti0019985* is associated with *CG18446* upregulation in three of the four backgrounds analyzed (Fig 6A). In the majority of strains, *roo*_*+7*_, *roo*_*-90*_, and *roo*_*-44*_ are not associated with changes in *CG18446* expression level (Fig 6B–6D). However, we can not discard that the presence of these insertions is associated with changes in the expression of *CG18446* in other developmental stages and/or in tissues not analyzed in this work.

### *FBti0019985* is associated with increased viability in nonstress and in cold-stress conditions

**Fig 7:**
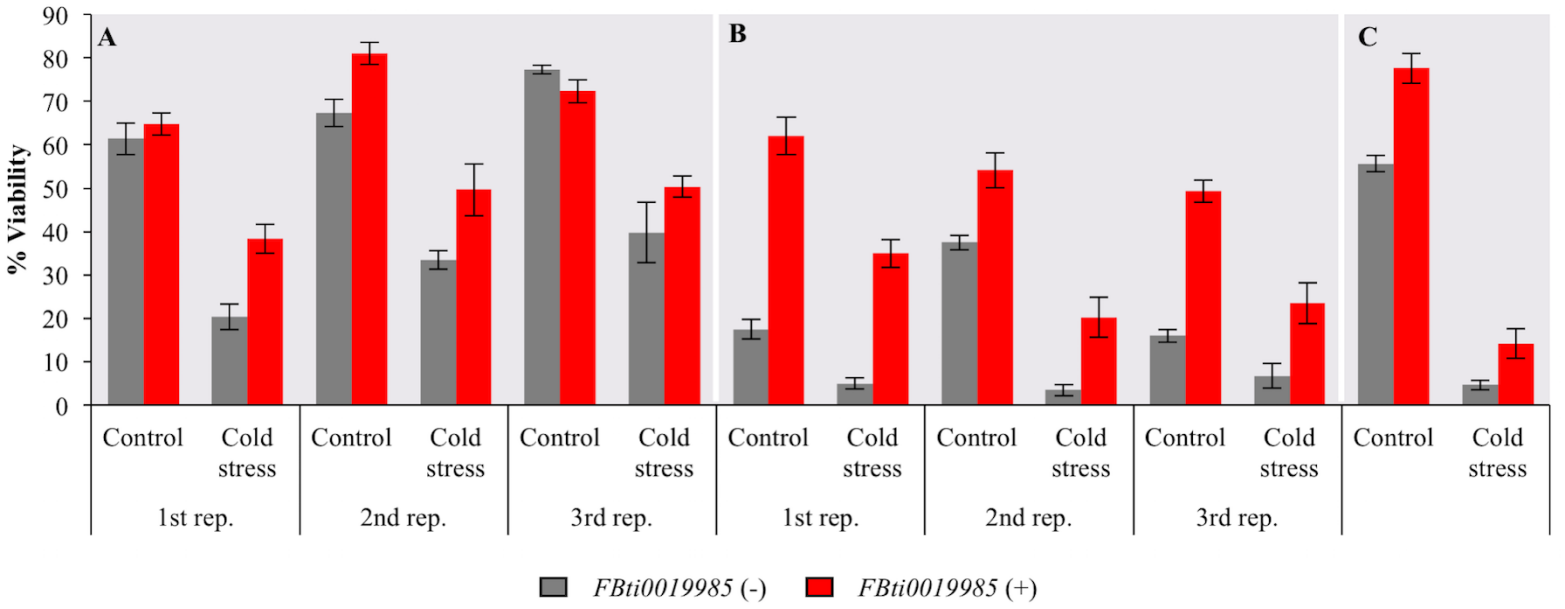
Flies with *FBti0019985* showed increased egg-to-adult viability under nonstress and under cold-stress conditions in three different genetic backgrounds. Egg-to-adult viability of strains without *FBti0019985* (grey) and with the *FBti0019985* insertion (red) in nonstress (control) and in cold-stress conditions. Results of the three replicates performed with (A) *RAL-783* and *RAL-810*, (B) *RAL-908* and *RAL-802*, and (C) *IV22* and *IV68*. Error bars represent the SEM of the different vials analyzed in each experiment.

We have shown that *FBti0019985* affects the transcript length and it is associated with upregulation of *CG18446* in most of the genetic backgrounds analyzed (Figs 5 and 6A). Because *CG18446* has been previously identified as a cold-stress candidate gene, we tested whether flies with and without *FBti0019985* differed in their sensitivity to cold-stress [21]. We first compared *RAL-810*, which carries *FBti0019985*, with *RAL-783*, which does not carry any of the nine *roo* insertions (Fig 7A). We performed three biological replicates. ANOVA analyses showed that the experimental condition (nonstress or cold-stress) and the insertion genotype (presence or absence of *FBti0019985*) were significant (Table 2). Flies with *FBti0019985* had a higher viability than flies without this insertion in both nonstress and cold-stress conditions. Furthermore, the interaction between these two factors was also significant suggesting that the effect of the insertion is larger in cold-stress conditions (Fig 7A and Table 2).

**Table 2:** ANOVA for cold-stress assays in flies with and without different *roo* solo-LTR insertions.

Two-way ANOVA							
		Experimental condition		Insertion genotype		Experimental condition * Insertion genotype	
Insertion	Strains	P-value	Effect size^a^	P-value	Effect size^a^	P-value	Effect size^a^
***FBti0019985***	*RAL-810 (FBti0019985) /*	≪0.001	0.69	≪0.001	0.13	0.034	0.04
	*RAL-783 (roo-)*						
	*RAL-802 (FBti0019985) /*	≪0.001	0.56	≪0.001	0.57	0.932	-
	*RAL-908 (roo-)*						
	*IV68 (FBti0019985) /*	≪0.001	0.74	0.003	0.25	0.981	-
	*IV22 (roo-)*						
***roo***_***+7***_	*RAL-405 (roo*_*+7*_*) /*	≪0.001	0.75	0.001	0.59	0.497	-
	*RAL-783 (roo-)*						
	*RAL-911 (roo*_*+7*_*) /*	≪0.001	0.76	0.530	-	0.220	-
	*RAL-783 (roo-)*						
***roo***_***-90***_	*RAL-21 (roo*_*-90*_*) /*	≪0.001	0.88	0.358	-	0.118	-
	*RAL-783 (roo-)*						
	*RAL-820 (roo*_*-90*_*) /*	≪0.001	0.71	0.681	-	0.123	-
	*RAL-783 (roo-)*						
***roo***_***-44***_	*RAL-195 (roo*_*-44*_*) /*	≪0.001	0.79	0.038	0.31	0.027	0.35
	*RAL-783 (roo-)*						
	*RAL-383 (roo*_*-44*_*) /*	≪0.001	0.95	≪0.001	0.76	0.991	-
	*RAL-783 (roo-)*						
***roo***_***-68***_	*RAL-75 (roo*_*-68*_*) /*	≪0.001	0.66	0.505	-	0.004	0.51
	*RAL-783 (roo-)*						
	*RAL-716 (roo*_*-68*_*) /*	≪0.001	0.87	0.002	0.56	0.032	0.33
	*RAL-783 (roo-)*						

^a^Partial eta-squared values calculated as a measure of effect size.

We repeated the experiment using flies with different genetic backgrounds: *RAL-802* that carries *FBti0019985* and *RAL-908* that does not carry this insertion (Fig 7B). ANOVA analyses showed that the experimental condition and the insertion genotype are significant while the interaction between these two factors was not significant (Table 2). *RAL-802* flies had a higher egg-to-adult viability in nonstress and in cold-stress conditions compared with flies without *FBti0019985*.

Finally, we tested whether flies from a different population, *IV68* carrying *FBti0019985* and *IV22* without this particular insertion both collected in Italy, also showed significantly increased viability in nonstress and in cold-stress conditions (Fig 7C and Table 2). We found that *IV68* flies had a higher viability than flies without the *FBti0019985* insertion in both nonstress and cold-stress conditions (Table 2).

Overall, we found consistent results, across genetic backgrounds from two different natural populations, suggesting that flies with the *FBti0019985* insertion are associated with increased viability compared to flies without this insertion in nonstress and in cold-stress conditions. In all cases, the effect of the presence of the insertion was either medium or large (Table 2). In one of the genetic backgrounds, the effect was larger under cold-stress conditions (Fig 7A) while no interaction between experimental condition and insertion genotype was found in the other two backgrounds (Fig 7B and 7C).

### Other *roo* solo-LTR insertions in the proximal promoter of *CG18446* are not consistently associated with cold-stress phenotypes

**Fig 8:**
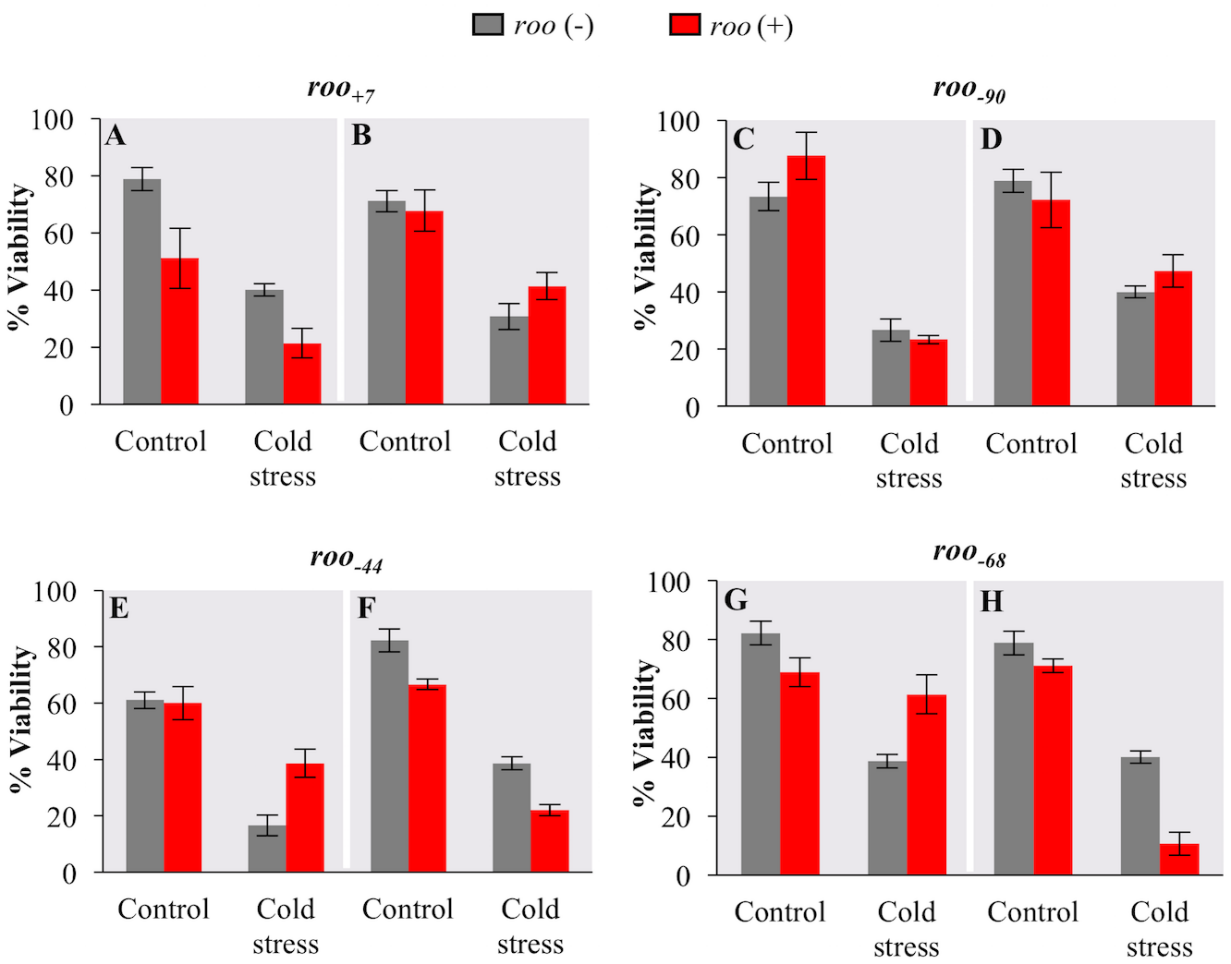
Other *roo* solo-LTR insertions are not consistently associated with cold-stress resistant phenotypes. Egg-to-adult viability in nonstress (control) and in cold-stress conditions of the *RAL-783* strain without any of the nine *roo* insertions (grey) and of different strains with *roo* insertions (red). (A) *RAL-405* (*roo*_*+7*_), (B) *RAL-911* (*roo*_*+7*_), (C) *RAL-21* (*roo*_*-90*_), (D) *RAL-820* (*roo*_*-90*_), (E) *RAL-195* (*roo*_*-44*_), (F) *RAL-383* (*roo*_*-44*_), (G) *RAL- 75* (*roo*_*-68*_), and (H) *RAL-716* (*roo*_*-68*_). Error bars represent the SEM of the different vials analyzed in each experiment.

We further checked whether another four *roo* solo-LTR insertions described in this work are associated with cold-stress phenotypes. For each insertion, we compared the egg-to-adult viability of flies with two different genetic backgrounds with the egg-to-adult viability of *RAL-783* that does not carry any of these insertions (Fig 8). In all cases, we performed ANOVA analyses to check whether the experimental conditions, insertion genotype, and/or the interaction between these two factors were significant (Table 2).

We found that the experimental condition had a significant effect on egg-to-adult viability in all the strains tested (Table 2). On the other hand, the effect of the insertion was only significant in some of the genetic backgrounds (Table 2). Among strains that carry the *roo*_*+7*_ insertion, the insertion genotype had an effect only in one of the two backgrounds tested (Fig 8A and 8B and Table 2). *RAL-405* flies with *roo*_*+7*_ insertion showed decreased viability (Fig 8A and Table 2). The presence/ absence of *roo*_*-90*_ did not have a significant effect on egg-to-adult viability (Fig 8C and 8D and Table 2). For *roo*_*-44*,_ while the insertion genotype had a significant effect on the two backgrounds tested, results were not consistent. In one background, the presence of the insertion is associated with increased viability under cold-stress conditions and the interaction between the treatment and the insertion genotype is significant (Fig 8E and Table 2), while in the other background the presence of *roo*_*-44*_ is associated with decreased viability (Fig 8F and Table 2). Finally, the presence of *roo*_*-68*_ significantly affected viability in only one of the two backgrounds tested: *RAL-716* flies carrying *roo*_*-68*_ showed decreased viability (Fig 8H and Table 2).

Overall our results suggested that the presence of *roo*_*+7*_, *roo*_*-90*_, *roo*_*-44*_, and *roo*_*-68*_ solo-LTR insertions reported in this work was not consistently associated with cold-stress phenotypes (Fig 8). These other insertions could have no phenotypic effect or could be involved in phenotypes not analyzed in this work.

### Inference of selection in the region flanking the *FBti0019985* insertion

We looked for evidence of positive selection in the 2 kb region flanking the *FBti0019985* insertion. We analyzed the number of segregating sites (*S*) in this region and estimated Tajima´s D, *iHS*, *nSL*, *H*_*12*_ and *XP-EHH* (see Material and Methods). We found reduced diversity in the strains with *FBti0019985*: the number of segregating sites in this region is significantly smaller than the number of segregating sites found in 2 kb regions of chromosome 2R, where the *FBti0019985* insertion is located (p-value = 0.015) (S4 Table). We also found that Tajima’s D was significantly negative in the 2 kb region where *FBti0019985* is inserted, as expected if this region is under positive selection (p-value = 0.009) (S4 Fig and S4 Table). Finally, we also found significant values of *iHS* and *H*_*12*_ in the region flanking the *FBti0019985* insertion (*p-value =* 0.048 and *p-value =* 0.023, respectively) (S5 Fig and S4 Table).

We also looked for evidence of selection taking into account not only the strains in which *FBti0019985* is inserted, but all the strains that contain one of the nine *roo* insertions described in this work. In this case, only *iHS* showed a marginally significant value (*p-value =* 0.049) (S6 Fig).

Overall, our results suggest that the strains carrying *FBti0019985* might be evolving under positive selection while the evidence for positive selection taking into account all the strains with one of the nine *roo* solo-LTRs, was only marginally significant.

## Discussion

Besides *FBti0019985*, we have discovered eight other *roo* solo-LTR elements inserted in the 368 bp region nearby the TSS of the cold-stress response gene *CG18446* (Fig 2) [21]. Each strain contained a single *roo* insertion and the population frequency of the different individual insertions varies from 1% to 17% (Fig 2). Full-length elements from the *roo* family are 8.7 kb long. Such long insertions in the proximal promoter of *CG18446* located in the first intron of *cbx*, might be deleterious, which could explain why all the identified insertions were solo-LTR elements. In *D*. *melanogaster*, repeated insertions of TEs have only been described in the proximal promoters of a particular gene class: *hsp* genes [50]. The susceptibility of *hsp* genes to TE insertions was attributed to their peculiar chromatin architecture: constitutively decondensed chromatin and nucleosome-free regions [51, 52]. However, promoter regions of non-*hsp* genes with similar chromatin architecture are not targets for TE insertions suggesting that chromatin accessibility is not sufficient to explain the susceptibility of *hsp* genes to TE insertions [50]. From a functional point of view, the presence of TEs in the promoter regions of *hsp* genes has been suggested to allow a rapid gene expression response to unpredictable temperature changes [50]. Similarly, the presence of *roo* insertions in the promoter of *CG18446* could also be enhancing the ability of this gene to respond to environmental challenges, although only one of the nine *roo* insertions was associated with cold-stress tolerance (see below). Interestingly, almost 100% of the insertions described in heat-shock genes are *P-element* insertions, and all the insertions described here are *roo* elements. *P-elements* preferentially insert in the 5' end of genes where they recognize a structural motif rather than a sequence motif [38, 39]. While 81% of *P-elements* insert in 5’ gene regions, our results showed that only 15.2% of the *roo* elements annotated in the reference genome are inserted in 5’ gene regions. Thus, with the data currently available, *roo* insertions do not seem to preferentially insert into 5’ gene regions although analyses of *de novo* insertions should shed more light on this issue.

Our results showed that the different *roo* elements inserted in the proximal promoter of *CG18446* differ in their molecular and functional effects (Table 3). We found that the two insertions that are more closely located to *CG18446*, *FBti0019985* and *roo*_*+7*_, provided an alternative TSS to this gene (Fig 5 and Table 3). However, only *FBti0019985* is associated with upregulation of *CG18446* expression (Fig 6 and Table 3). Besides providing an alternative TSS, the effect of the *FBti0019985* insertion on *CG18446* expression could be due to the addition of new regulatory regions (Fig 3A), to the disruption of the spacing of pre-existing ones (Fig 3B), and/or to the recruitment of HP1a protein that could also lead to changes in the expression of *CG18446* (Fig 4B). Finally, we cannot discard that polymorphisms other than the presence/absence of the *FBti0019985* insertion also affect the expression of *CG18446*.

**Table 3:** Summary of the experimental results obtained in fly strains with five different *roo* solo-LTR insertions.

Insertion	Orientation	Strain	5'-RACE	*CG18446* expression	Effect of the insertion in egg-to-adult viability
***FBti0019985***	5' to 3'	*RAL-810*	TSS inside TE	Upregulation	Increase
		*RAL-802*	-	No differences	Increase
		*IV68*	-	Upregulation	Increase
		*RAL-639*	-	Upregulation	-
***roo***_***+7***_	5' to 3'	*RAL-405*	TSS inside TE	Downregulation	Decrease
		*RAL-911*	-	No differences	No differences
		*RAL-887*	-	No differences	-
***roo***_***-44***_	3' to 5'	*RAL-195*	TSS outside TE	No differences	Increase
		*RAL-383*	TSS outside TE	No differences	Decrease
***roo***_***-68***_	3' to 5'	*RAL-75*	-	-	No differences
		*RAL-716*	-	-	Decrease
***roo***_***-90***_	5' to 3'	*RAL-21*	TSS outside TE	Upregulation	No differences
		*RAL-820*	-	No differences	No differences
		*RAL-88*	-	No differences	-

We found that the *FBti0019985* insertion, which is associated with increased *CG18446* expression, is consistently associated with increased viability in nonstress and in cold-stress conditions (Fig 7 and Table 3). Although we cannot exclude that other variants linked to *FBti0019985* contribute to the increased viability phenotypes, we argue that it is unlikely that the association between the *FBti0019985* insertion and increased viability in three different genetic backgrounds from two different natural populations would occur spuriously [53]. These results also suggest that *CG18446* is likely to play a role in cold tolerance as was previously suggested based on cold-stress selection experiments in which this gene was found to be overexpressed [21]. However, *FBti0019985* is present in only 10% of the out-of-Africa natural strains analyzed in this work. Our screening was focused on three out-of-Africa populations, thus we cannot discard that *FBti0019985* is present at higher frequencies in other populations. Alternatively, it is also possible that the relatively low frequency of *FBti0019985* is due to negative fitness effects of this insertion on other phenotypes. Cold-stress resistance has been associated with decreased starvation resistance [54, 55] and reduced fecundity [56, 57]. Therefore, the benefit of flies carrying *FBti0019985* in cold-stress conditions might be a cost, for example, when food resources are scarce.

While *FBti0019985* has a consistent cold-stress tolerance phenotype, four other *roo* insertions also located on the proximal promoter of *CG18446* did not (Fig 8 and Table 3). The insertion that is present at higher frequencies in out-of-Africa populations is *roo*_*-90*_ (Fig 2). However, this insertion is not associated with changes of expression of *CG18446* in embryos (Fig 6) and was not found to be associated with cold-stress tolerance phenotypes (Fig 8C and 8D and Table 3). It could be that this insertion has no phenotypic effect. Alternatively, *roo*_*-90*_ could be affecting a phenotype other than cold tolerance. A recent update in FlyBase revealed that *CG18446* is also an ethanol-regulated gene that could contribute to ethanol sensitivity or tolerance [58]. Another possibility is that *roo*_*-90*_ affects *cbx*. As the other *roo* insertion described in this work and *CG18446* gene, *roo*_*-90*_ is inserted in the first intron of *cbx* which has been functionally classified as a defense response to bacterium and spermatogenesis gene [59] (Fig 1). Elucidating whether *roo*_*-90*_ has an adaptive effect is beyond the scope of this paper.

Overall, we did not find evidence of positive selection at the DNA level in the region where the nine *roo* solo-LTR elements are inserted. We did find evidence of reduced diversity in this region when only the strains containing *FBti0019985* were considered (S4–S6 Figs and S4 Table). Further analyses with a bigger dataset of strains is needed in order to determine whether this region shows signals of positive selection at the DNA level.

In summary, our results showed that different TE insertions in the same gene promoter region might have different molecular and functional consequences. Thus, the description of complex regions, as the one reported in this work, should be followed by functional analysis of the structural variants if we want to elucidate which ones are functionally relevant.

## Materials and Methods

### Fly stocks

We used inbred strains from the Drosophila Genetic Reference Panel (DGRP [31, 32]) and isofemale strains from an Italian population collected in Castellana Grotte (Bari, Italy [33]) to perform the molecular and phenotypic assays.

### Analysis of presence/absence by PCR of the nine solo-LTR *roo* insertions

We used a PCR approach to check for presence/ absence of *FBti0019985* in 28 strains from the North Carolina population and in 15 strains from Italy. The primers used were FBti0019985_FL (5’-GGCATCATAAAACCGTTGAACAC-3’), FBti0019985_L (5’-AGTCCCTTAGTGGGAGACCACAG-3’) and FBti0019985_R (5’-CGTAGGATCAGTGGGTGAAAATG-3’) (Fig 1). Primers FBti0019985_L and FBti0019985_R are expected to give a 616 bp band when the TE is present. Primers FBti0019985_FL and FBti0019985_R are expected to give a 638 bp band when the TE is absent and a 1066 bp band when the TE is present. All PCR bands giving evidence of presence and some of the PCR bands giving evidence of absence were cloned using TOPO TA Cloning Kit for Sequencing (Invitrogen) following the manufacturer’s instructions and Sanger-sequenced using M13 forward and/or M13 reverse primers to verify the results. Sequences have been deposited in GenBank under accession numbers KU672690-KU672720.

### Analysis of the population frequencies of the nine *roo* solo-LTR insertions using *Tlex2*

We estimated the frequencies of the nine *roo* solo-LTR insertions described in this work using *T-lex2* software [34]. Because *T-lex2* works only for annotated TEs, we constructed eight new reference sequences including each one of the newly described *roo* solo-LTR insertions. The new reference sequences included 500 bp at each side of the TE and the TSD of each insertion.

We run *T-lex2* in strains from three different populations: 50 strains from North Carolina (DGRP [31, 32]), 27 strains from a population collected in Stockholm, Sweden [33], and 67 strains from a population collected in Siavonga, Zambia [60]. As a control, we also run *T-lex2* in the strains for which we have PCR results (S1 Table). We obtained results for 21 out of 50 DGRP strains, 26 out of 27 Swedish strains and 42 out of 67 Zambian strains. In some of the strains, *T-lex2* detects more than one insertion per strain. However, PCR analyses of these strains revealed that only one insertion was present. These results suggest that *T-lex2* cannot accurately estimate the frequency of insertion when they are closely located to each other. We thus discarded *T-lex2* results indicating the presence of more than one insertion per strain. Other factors such as the quality of the reads and the coverage of the different strains could also be affecting *T-lex2* results.

### Analysis of target site motifs

Target site motifs were constructed in WebLogo (http://weblogo.berkeley.edu) using six TSDs sequences obtained in this work and 41 TSDs sequences predicted with *T-lex2* software [34].

### Phylogenetic analysis

For each *roo* solo-LTR insertion, we constructed a consensus sequence taking into account the 26 strains sequenced in this work using *Sequencher* 5.0 software. We aligned the nine *roo* insertion consensus sequences with 115 of the 137 other *roo* insertions present in the *D*. *melanogaster* genome using the multiple sequence aligner program MAFFT [61]. The quality sequence of the other 22 *roo* insertions was too low to include them in the alignment. A maximum likelihood tree was inferred using RAxML Version 8 [62] under the general time-reversible nucleotide model and a gamma distribution of evolutionary rates. We use the ETE toolkit Python framework for the analysis and visualization of trees [63].

### *roo* insertions and *CG18446* promoter sequence analysis

We looked for conservation of the Transcription Factor Binding Sites (TFBSs) previously described in the *roo* family [8] in all the *roo* solo-LTRs characterized in this work. First, we downloaded from FlyBase version r6.06 (http://flybase.org) the *fasta* file of *FBti0019985* sequence (genome region 2R: 9,871,090–9,871,523). We also searched for TFBSs in the *roo* insertions and in the *CG18446* promoter regions using all the available JASPAR CORE Insecta matrices (http://jaspar.genereg.net). Only those sites predicted with a relative score higher than 0.995 were considered. We identified four new TFBS in *FBti0019985* sequence: *Deaf1*, *ara*, *mir*, and *caup*. We then look for conservation of the identified motifs in all the *roo* solo-LTR sequences described in this work. For some strains, we used the information available in http://popdrowser.uab.cat [64].

### Detection of piRNA reads

We used three piRNA libraries [44–46] to map piRNA reads to a 1.4 kb region including *FBti0019985* and to all the *roo* insertions described in this work following the methodology described in Ullastres *et al* (2015) [33]. Briefly, we used BWA-MEM package version 0.7.5 a-r405 [65] to align the reads and then we used SamTools and BamTools [66] to index and filter by sense/antisense reads. The total read density was obtained using R (Rstudio v0.98.507) [67].

### Detection of HP1a binding sites

We used modENCODE ChIP-Seq data [47] to map HP1a reads to a 1.4 kb region including *FBti0019985* and to all the *roo* insertions described in this work following the methodology described in Ullastres *et al* (2015) [33]. We aligned the reads using BWA-MEM package version 0.7.5 a-r405 [65]. The total read density was obtained using R (Rstudio v0.98.507) [67].

### 5’-RACE experiments

5-to-7 day-old flies were placed in a fly cage with egg-laying medium (2% agar with apple juice and a piece of fresh yeast) during 4 hours. Then, adult flies were separated and embryos were collected following the suspension method described in Schou (2013) [68]. Embryo dechorionation was done by bleach (50%) immersion. Total RNA was extracted using TRIzol Plus RNA Purification Kit (Ambion). RNA was then treated on-column with DNase I (Thermo) during purification, and then treated once more after purification. 5’-RACE was performed with FirstChoice RLM-RACE Kit and using Small-scale reaction RNA processing with RNA samples of *RAL-783* (*roo*-), *RAL-810* (*FBti0019985*), *RAL-405* (*roo*_+7_), *RAL-21* (*roo*_-90_), *RAL-383* (*roo*_-44_) and *RAL-195* (*roo*_-44_). The gene specific outer primer was 5’-GACACTCTTCGGTTGGTGGA-3’ and the gene specific inner primer was 5’-ACAACTGTTCTGTAGGATCGC-3’. The control primer was 5’-TAGTCCGCAGAGAAACGTCG-3’. Inner PCR products were then cloned and Sanger-sequenced as mentioned above. Sequences have been deposited in GenBank under accession numbers KU672721-KU672722.

### Quantitative RT-PCR expression analysis

Embryo collection and RNA extraction was performed as described before. Reverse transcription was carried out using 500 ng of total RNA using Transcriptor First Strand cDNA Synthesis Kit (Roche). The cDNA was then used in a 1/50 dilution for qRT-PCR with SYBR green master-mix (Bio-Rad) on an iQ5 Thermal cycler. *CG18446* expression was measured using specific primers (5’-GAGCAGTTGGAATCGGGTTTTAC-3’ and 5’-GTATGAATCGCAGTCCAGCCATA-3’) spanning 99 bp cDNA in the exon 1/exon 2 junction of *CG18446*. The primer pair efficiency was 99,1% (r^2^ larger than 0.99). *CG18446* expression was normalized with *Act5C* expression levels (5’-GCGCCCTTACTCTTTCACCA-3’ and 5’-ATGTCACGGACGATTTCACG-3’).

### Cold-stress resistance assays

Embryo collection was performed as mentioned above. Embryos were put into 50 ml fresh food vials. When embryos were 4–8 hour-old, they were kept at 1 C for 14 hours and then they were kept at room temperature (22–25 C). Simultaneously, control vials were always kept at room temperature (22–25 C) and never exposed to cold-stress. A total of 8–20 vials were analyzed per experiment. The same number of embryos per vial, 30 or 50, were used for all the replicates of a given experiment. Percentage viability was calculated based on the number of emerged flies to the total number of embryos placed in each vial.

Statistical significance was calculated performing two-way ANOVA using SPSS v21. We combined all the data into a full model: experimental condition (stress and nonstress), insertion genotype (presence/absence of the insertion) and interaction between these two factors. For those experiments in which more than one replicate was performed, the replicate effect was also taken into account. Because our dependent variable was a proportion, we used the arcsine transformation of the data before performing statistical analysis. We tested whether the data was normally distributed using Kolmogorov-Smirnov test. When the data was not normally distributed after the arcsine transformation, we applied the rank transformation. When the statistical test was significant, we estimated partial eta-squared values as a measure of the effect size (0.01 small effect, 0.06 medium effect, and 0.14 large effect).

### Inferences of selection in the region flanking the *roo* solo-LTR insertions

We estimated the number of segregating sites (*S*), Tajima´s D, *iHS*, *nSL* and *XP-EHH* in the 2 kb region flanking the *FBti0019985* insertion (chromosome 2R: 5758000–5760000) in 10 DGRP strains containing this insertion, in the 23 DGRP strains containing one of the *roo* insertions described in this work, and in the 15 strains that do not contain any insertion in the promoter region of *CG18446*. Note that the coordinates of *FBti0019985* in the r5 of the *D*. *melanogaster* genome used by the DGRP project to generate the *vcf* files are 2R: 5,758,595–5,759,028. *S* and Tajima´s D are standard mesures of neutrality. *iHS* and *nSL* tests identify hard sweeps although they have some power to detect soft sweeps as well [69, 70]. *H*_*12*_ tests for positive selection on new variation and standing genetic variation within a population, that is, it searches both for soft and hard sweeps in a population [71]. Finally, *XP-EHH* is a statistical test of positive selection in one population that uses between populations comparisons to increase power in regions near fixation in the selected population [72].

We have used *vcftools* to calculate the number of segregating sites, and Tajima´s D using parameters –maf 1/(2*n*), where *n* is the sample size, and –remove-indels. We have obtained *iHS*, *nSL*, and *XP-EHH* using the *selscan* software with default parameters [73]. Finally, we have calculated *H*_*12*_ with *ad hoc* scripts. The four latter statistics require phased data. Thus, chromosome 2R of the 205 DGRP strains were phased together using ShapeIt [74].

To calculate the significance for the number of segregating sites, we resampled at random the same number of strains from the 205 DGRP strains available and calculated the distribution of segregating sites in the same 2 kb region. To calculate the significance of Tajima´s D, *iHS*, *nSL* and *XP-EHH*, we have used the empirical distributions of these statistics obtained from chromosome 2R.

## Supporting Information

S1 Fig *roo* consensus Target Site Duplication (TSD). A frequency plot was built with all the TSD identified in this work, except the TSD of *roo*_*-19*_ and *roo*_*+7*_ that had four and two nucleotides instead of five, respectively, and with the 41 *roo* TSD motifs identified by Fiston-Lavier *et al* (2015) [34] (see Materials and Methods).
(TIFF)

S2 Fig Phylogenetic tree including the nine *roo* elements analyzed in this work and the 115 *roo* elements annotated in the *D*. *melanogaster* reference genome. The nine *roo* elements sequenced in this work are depicted in red.
(TIFF)

S3 Fig Sequence alignments of the regulatory regions identified in *roo* insertions and in the *CG18446* promoter region. Single nucleotide polymorphisms are highlighted in red. (A) Alignment of the different *roo* insertions analyzed in this work. For *RAL-502* and *RAL-857* we could only sequence a partial region of the insertion and thus we only analyzed the *Inr* motif. (B) Alignment of the three regions with matrix association potential. (C) Alignment of the *CG18446* promoter region in the different strains analyzed. Underlined sequences are from popdrowser [64]. For additional details see Fig 3 legend.
(PDF)

S4 Fig From top to bottom: Tajima’s D in the 23 strains with one of the nine solo-LTR insertions, Tajima’s D in the 10 strains with the *FBti0019985* insertion, and Tajima’s D in the 15 strains without any of the nine insertions. (PDF)

S5 Fig From top to bottom, results for *XP-EHH*, *H*_*12*,_ *nSL*, and *iHS*. *H*_*12*_ was calculated on haplotypes of 40 segregating sites. All results are for the 10 strains with the *FBti0019985* insertion combined with the 15 strains without any of the nine insertions, except for *XP-EHH*, which is calculated between the 10 strains with the *FBti0019985* insertion and the 15 strains without any of the nine insertions. Horizontal dashed lines show significance levels while vertical dashed lines show the region of the insertion.
(PDF)

S6 Fig Results for *XP-EHH*, *H*_*12*,_ *nSL*, and *iHS* from top to bottom calculated with the 23 strains that contain one of the nine *roo* insertions and the 15 strains without any of the *roo* insertions. See legend of S5 Fig for details.
(PDF)

S1 Table Allele frequency estimates using *T-lex2* for the nine *roo* solo-LTR insertions analyzed. (A) Summary of the *T-lex2* results in all populations. (B) Results for DGRP population. (C) Results for Sweden population. (D) Results for Zambia population. (E) Results for the Italian population for which PCRs were also performed. (F) Results for the DGRP strains for which PCR were also performed.
(XLSX)

S2 Table Sequence alignments of the cis-regulatory motifs located in *roo* solo-LTR insertions and in the *CG18446* promoter region. (A) Transcription factor binding sites and promoter motifs, and (B) Matrix Associated regions, found in *FBti0019985*. (C) Transcription factor binding sites and promoter motifs found in the *CG18446* promoter region.
(DOCX)

S3 Table Number of piRNA reads (A) and HP1a reads (B) mapping to each one of the nine *roo* insertions analyzed in this work. The total number of piRNA reads and of HP1a reads per nucleotide position, and the average number of piRNA reads and of HP1a reads per insertion are given.
(XLSX)

S4 Table Results of the different statistics used to infer positive selection in the region flanking the nine solo-LTR insertions. (DOCX)

S1 Text Phylogenetic tree containing the nine *roo* elements sequenced in this work and the 115 *roo* elements annotated in the *D*. *melanogaster* reference genome. (TXT)

## References

[1] Juven-Gershon T, Kadonaga JT. Regulation of gene expression via the core promoter and the basal transcriptional machinery. Developmental biology. 2010;339(2):225–9. 10.1016/j.ydbio.2009.08.009 19682982 PMC2830304

[2] Haberle V, Lenhard B. Promoter architectures and developmental gene regulation. Semin Cell Dev Biol. 2016. 10.1016/j.semcdb.2016.01.014 26783721

[3] Hoskins RA, Landolin JM, Brown JB, Sandler JE, Takahashi H, Lassmann T, et al Genome-wide analysis of promoter architecture in Drosophila melanogaster. Genome Res. 2011;21(2):182–92. 10.1101/gr.112466.110 21177961 PMC3032922

[4] Carninci P, Sandelin A, Lenhard B, Katayama S, Shimokawa K, Ponjavic J, et al Genome-wide analysis of mammalian promoter architecture and evolution. Nat Genet. 2006;38(6):626–35. 16645617 10.1038/ng1789

[5] Kawaji H, Frith MC, Katayama S, Sandelin A, Kai C, Kawai J, et al Dynamic usage of transcription start sites within core promoters. Genome Biol. 2006;7(12):R118 17156492 10.1186/gb-2006-7-12-r118 PMC1794431

[6] Consortium Fantom, Suzuki H, Forrest AR, van Nimwegen E, Daub CO, Balwierz PJ, et al The transcriptional network that controls growth arrest and differentiation in a human myeloid leukemia cell line. Nat Genet. 2009;41(5):553–62. 10.1038/ng.375 19377474 PMC6711855

[7] Rach EA, Winter DR, Benjamin AM, Corcoran DL, Ni T, Zhu J, et al Transcription initiation patterns indicate divergent strategies for gene regulation at the chromatin level. PLoS genetics. 2011;7(1):e1001274 10.1371/journal.pgen.1001274 21249180 PMC3020932

[8] Batut P, Dobin A, Plessy C, Carninci P, Gingeras TR. High-fidelity promoter profiling reveals widespread alternative promoter usage and transposon-driven developmental gene expression. Genome Res. 2013;23(1):169–80. 10.1101/gr.139618.112 22936248 PMC3530677

[9] McClintock B. Intranuclear systems controlling gene action and mutation. Brookhaven Symp Biol. 1956;2(8):58–74. 13293421

[10] Britten RJ, Davidson EH. Gene regulation for higher cells: a theory. Science. 1969;165(3891):349–57. 5789433 10.1126/science.165.3891.349

[11] Guio L, Barron MG, Gonzalez J. The transposable element Bari-Jheh mediates oxidative stress response in Drosophila. Molecular ecology. 2014;23(8):2020–30. 10.1111/mec.12711 24629106

[12] Guio L, Gonzalez J. The dominance effect of the adaptive transposable element insertion Bari-Jheh depends on the genetic background. Genome biology and evolution. 2015;7(5):1260–6. 10.1093/gbe/evv071 25912044 PMC4453066

[13] Chuong EB, Elde NC, Feschotte C. Regulatory evolution of innate immunity through co-option of endogenous retroviruses. Science. 2016;351(6277):1083–7. 10.1126/science.aad5497 26941318 PMC4887275

[14] Lerman DN, Feder ME. Naturally occurring transposable elements disrupt hsp70 promoter function in Drosophila melanogaster. Molecular biology and evolution. 2005;22(3):776–83. 15574805 10.1093/molbev/msi063

[15] Gonzalez J, Lenkov K, Lipatov M, Macpherson JM, Petrov DA. High rate of recent transposable element-induced adaptation in Drosophila melanogaster. PLoS biology. 2008;6(10):e251 10.1371/journal.pbio.0060251 18942889 PMC2570423

[16] Bronner G, Taubert H, Jackle H. Mesoderm-specific B104 expression in the Drosophila embryo is mediated by internal cis-acting elements of the transposon. Chromosoma. 1995;103(10):669–75. 7664613 10.1007/BF00344227

[17] Scherer G, Telford J, Baldari C, Pirrotta V. Isolation of cloned genes differentially expressed at early and late stages of Drosophila embryonic development. Developmental biology. 1981;86(2):438–47. 6269930 10.1016/0012-1606(81)90202-5

[18] Scherer G, Tschudi C, Perera J, Delius H, Pirrotta V. B104, a new dispersed repeated gene family in Drosophila melanogaster and its analogies with retroviruses. J Mol Biol. 1982;157(3):435–51. 6181263 10.1016/0022-2836(82)90470-3

[19] Meyerowitz EM, Hogness DS. Molecular organization of a Drosophila puff site that responds to ecdysone. Cell. 1982;28(1):165–76. 6279311 10.1016/0092-8674(82)90386-5

[20] FitzGerald PC, Sturgill D, Shyakhtenko A, Oliver B, Vinson C. Comparative genomics of Drosophila and human core promoters. Genome Biol. 2006;7(7):R53 16827941 10.1186/gb-2006-7-7-r53 PMC1779564

[21] Telonis-Scott M, Hallas R, McKechnie SW, Wee CW, Hoffmann AA. Selection for cold resistance alters gene transcript levels in Drosophila melanogaster. Journal of insect physiology. 2009;55(6):549–55. 10.1016/j.jinsphys.2009.01.010 19232407

[22] Hoffmann AA, Scott M, Partridge L, Hallas R. Overwintering in Drosophila melanogaster: outdoor field cage experiments on clinal and laboratory selected populations help to elucidate traits under selection. J Evol Biol. 2003;16(4):614–23. 14632225 10.1046/j.1420-9101.2003.00561.x

[23] Kellermann V, Loeschcke V, Hoffmann AA, Kristensen TN, Flojgaard C, David JR, et al Phylogenetic constraints in key functional traits behind species' climate niches: patterns of desiccation and cold resistance across 95 Drosophila species. Evolution. 2012;66(11):3377–89. 10.1111/j.1558-5646.2012.01685.x 23106704

[24] Goto SG. Expression of Drosophila homologue of senescence marker protein-30 during cold acclimation. Journal of insect physiology. 2000;46(7):1111–20. 10817837 10.1016/s0022-1910(99)00221-8

[25] Goto SG. A novel gene that is up-regulated during recovery from cold shock in Drosophila melanogaster. Gene. 2001;270(1–2):259–64. 11404024 10.1016/s0378-1119(01)00465-6

[26] Greenberg AJ, Moran JR, Coyne JA, Wu CI. Ecological adaptation during incipient speciation revealed by precise gene replacement. Science. 2003;302(5651):1754–7. 14657496 10.1126/science.1090432

[27] Qin W, Neal SJ, Robertson RM, Westwood JT, Walker VK. Cold hardening and transcriptional change in Drosophila melanogaster. Insect Mol Biol. 2005;14(6):607–13. 16313561 10.1111/j.1365-2583.2005.00589.x

[28] Morgan TJ, Mackay TF. Quantitative trait loci for thermotolerance phenotypes in Drosophila melanogaster. Heredity (Edinb). 2006;96(3):232–42. 16404413 10.1038/sj.hdy.6800786

[29] Hoffmann AA, Blacket MJ, McKechnie SW, Rako L, Schiffer M, Rane RV, et al A proline repeat polymorphism of the Frost gene of Drosophila melanogaster showing clinal variation but not associated with cold resistance. Insect Mol Biol. 2012;21(4):437–45. 10.1111/j.1365-2583.2012.01149.x 22708613

[30] Mamillapalli A, Pathak RU, Garapati HS, Mishra RK. Transposable element 'roo' attaches to nuclear matrix of the Drosophila melanogaster. Journal of insect science. 2013;13:111 10.1673/031.013.11101 24735214 PMC4011374

[31] Huang W, Massouras A, Inoue Y, Peiffer J, Ramia M, Tarone AM, et al Natural variation in genome architecture among 205 Drosophila melanogaster Genetic Reference Panel lines. Genome Res. 2014;24(7):1193–208. 10.1101/gr.171546.113 24714809 PMC4079974

[32] Mackay TF, Richards S, Stone EA, Barbadilla A, Ayroles JF, Zhu D, et al The Drosophila melanogaster Genetic Reference Panel. Nature. 2012;482(7384):173–8. 10.1038/nature10811 22318601 PMC3683990

[33] Ullastres A, Petit N, Gonzalez J. Exploring the Phenotypic Space and the Evolutionary History of a Natural Mutation in Drosophila melanogaster. Molecular biology and evolution. 2015;32(7):1800–14. 10.1093/molbev/msv061 25862139 PMC4476160

[34] Fiston-Lavier AS, Barron MG, Petrov DA, Gonzalez J. T-lex2: genotyping, frequency estimation and re-annotation of transposable elements using single or pooled next-generation sequencing data. Nucleic Acids Res. 2015;43(4):e22 10.1093/nar/gku1250 25510498 PMC4344482

[35] Attrill H, Falls K, Goodman JL, Millburn GH, Antonazzo G, Rey AJ, et al FlyBase: establishing a Gene Group resource for Drosophila melanogaster. Nucleic Acids Res. 2016;44(D1):D786–92. 10.1093/nar/gkv1046 26467478 PMC4702782

[36] Bernstein M, Lersh RA, Subrahmanyan L, Cline TW. Transposon Insertions Causing Constitutive Sex-Lethal Activity in Drosophila melanogaster Affect Sxl Sex-Specific Transcript Splicing. Genetics. 1995;139:631–48. 7713421 10.1093/genetics/139.2.631 PMC1206370

[37] Linheiro RS, Bergman CM. Whole genome resequencing reveals natural target site preferences of transposable elements in Drosophila melanogaster. PLoS One. 2012;7(2):e30008 10.1371/journal.pone.0030008 22347367 PMC3276498

[38] Spradling AC, Stern DM, Kiss I, Roote J, Laverty T, Rubin GM. Gene disruptions using P transposable elements: an integral component of the Drosophila genome project. Proceedings of the National Academy of Sciences of the United States of America. 1995;92(24):10824–30. 7479892 10.1073/pnas.92.24.10824 PMC40524

[39] Liao GC, Rehm EJ, Rubin GM. Insertion site preferences of the P transposable element in Drosophila melanogaster. Proceedings of the National Academy of Sciences of the United States of America. 2000;97(7):3347–51. 10716700 10.1073/pnas.050017397 PMC16242

[40] Reed DE, Huang XM, Wohlschlegel JA, Levine MS, Senger K. DEAF-1 regulates immunity gene expression in Drosophila. Proceedings of the National Academy of Sciences of the United States of America. 2008;105(24):8351–6. 10.1073/pnas.0802921105 18550807 PMC2448840

[41] Dantoft W, Davis MM, Lindvall JM, Tang X, Uvell H, Junell A, et al The Oct1 homolog Nubbin is a repressor of NF-kappaB-dependent immune gene expression that increases the tolerance to gut microbiota. BMC biology. 2013;11:99 10.1186/1741-7007-11-99 24010524 PMC3849502

[42] Sentmanat MF, Elgin SC. Ectopic assembly of heterochromatin in Drosophila melanogaster triggered by transposable elements. Proceedings of the National Academy of Sciences of the United States of America. 2012;109(35):14104–9. 10.1073/pnas.1207036109 22891327 PMC3435190

[43] Lee YC. The Role of piRNA-Mediated Epigenetic Silencing in the Population Dynamics of Transposable Elements in Drosophila melanogaster. PLoS genetics. 2015;11(6):e1005269 10.1371/journal.pgen.1005269 26042931 PMC4456100

[44] Li C, Vagin VV, Lee S, Xu J, Ma S, Xi H, et al Collapse of germline piRNAs in the absence of Argonaute3 reveals somatic piRNAs in flies. Cell. 2009;137(3):509–21. 10.1016/j.cell.2009.04.027 19395009 PMC2768572

[45] Satyaki PR, Cuykendall TN, Wei KH, Brideau NJ, Kwak H, Aruna S, et al The Hmr and Lhr hybrid incompatibility genes suppress a broad range of heterochromatic repeats. PLoS genetics. 2014;10(3):e1004240 10.1371/journal.pgen.1004240 24651406 PMC3961192

[46] Shpiz S, Ryazansky S, Olovnikov I, Abramov Y, Kalmykova A. Euchromatic transposon insertions trigger production of novel Pi- and endo-siRNAs at the target sites in the drosophila germline. PLoS genetics. 2014;10(2):e1004138 10.1371/journal.pgen.1004138 24516406 PMC3916259

[47] Kharchenko PV, Alekseyenko AA, Schwartz YB, Minoda A, Riddle NC, Ernst J, et al Comprehensive analysis of the chromatin landscape in Drosophila melanogaster. Nature. 2011;471(7339):480–5. 10.1038/nature09725 21179089 PMC3109908

[48] Eissenberg JC, Elgin SC. HP1a: a structural chromosomal protein regulating transcription. Trends Genet. 2014;30(3):103–10. 10.1016/j.tig.2014.01.002 24555990 PMC3991861

[49] dos Santos G, Schroeder AJ, Goodman JL, Strelets VB, Crosby MA, Thurmond J, et al FlyBase: introduction of the Drosophila melanogaster Release 6 reference genome assembly and large-scale migration of genome annotations. Nucleic Acids Res. 2015;43(Database issue):D690–7. 10.1093/nar/gku1099 25398896 PMC4383921

[50] Walser JC, Chen B, Feder ME. Heat-shock promoters: targets for evolution by P transposable elements in Drosophila. PLoS genetics. 2006;2(10):e165 17029562 10.1371/journal.pgen.0020165 PMC1592238

[51] Lerman DN, Michalak P, Helin AB, Bettencourt BR, Feder ME. Modification of Heat-Shock Gene Expression in Drosophila melanogaster Populations via Transposable Elements. Molecular biology and evolution. 2003;20(1):135–44. 12519916 10.1093/molbev/msg015

[52] Shilova VY, Garbuz DG, Myasyankina EN, Chen B, Evgen'ev MB, Feder ME, et al Remarkable site specificity of local transposition into the Hsp70 promoter of Drosophila melanogaster. Genetics. 2006;173(2):809–20. 16582443 10.1534/genetics.105.053959 PMC1526513

[53] Gruber JD, Genissel A, Macdonald SJ, Long AD. How repeatable are associations between polymorphisms in achaete-scute and bristle number variation in Drosophila? Genetics. 2007;175(4):1987–97. 17277365 10.1534/genetics.106.067108 PMC1855119

[54] Kenny MC, Wilton A, Ballard JWO. Seasonal trade-off between starvation resistance and cold resistance in temperate wild-caught Drosophila simulans. Australian Journal of Entomology. 2008;47(1):20–3.

[55] Hoffmann AA, Hallas R, Anderson AR, Telonis-Scott M. Evidence for a robust sex-specific trade-off between cold resistance and starvation resistance in Drosophila melanogaster. J Evol Biol. 2005;18(4):804–10. 16033551 10.1111/j.1420-9101.2004.00871.x

[56] Watson MJO, Hoffmann AA. Acclimation, cross-generation effects, and the response to selection for increased cold resistance in Drosophila. Evolution. 1996;50(3):1182–92. 28565271 10.1111/j.1558-5646.1996.tb02359.x

[57] Marshall KE, Sinclair BJ. Repeated stress exposure results in a survival-reproduction trade-off in Drosophila melanogaster. Proc Biol Sci. 2010;277(1683):963–9. 10.1098/rspb.2009.1807 19939842 PMC2842730

[58] Kong EC, Allouche L, Chapot PA, Vranizan K, Moore MS, Heberlein U, et al Ethanol-regulated genes that contribute to ethanol sensitivity and rapid tolerance in Drosophila. Alcohol Clin Exp Res. 2010;34(2):302–16. 10.1111/j.1530-0277.2009.01093.x 19951294 PMC2903447

[59] Ayres JS, Freitag N, Schneider DS. Identification of Drosophila Mutants Altering Defense of and Endurance to Listeria monocytogenes Infection. Genetics. 2008;178:1807–15. 10.1534/genetics.107.083782 18245331 PMC2278058

[60] Lack JB, Cardeno CM, Crepeau MW, Taylor W, Corbett-Detig RB, Stevens KA, et al The Drosophila genome nexus: a population genomic resource of 623 Drosophila melanogaster genomes, including 197 from a single ancestral range population. Genetics. 2015;199(4):1229–41. 10.1534/genetics.115.174664 25631317 PMC4391556

[61] Katoh K, Standley DM. MAFFT multiple sequence alignment software version 7: improvements in performance and usability. Molecular biology and evolution. 2013;30(4):772–80. 10.1093/molbev/mst010 23329690 PMC3603318

[62] Stamatakis A. RAxML version 8: a tool for phylogenetic analysis and post-analysis of large phylogenies. Bioinformatics. 2014;30(9):1312–3. 10.1093/bioinformatics/btu033 24451623 PMC3998144

[63] Huerta-Cepas J, Serra F, Bork P. ETE 3: Reconstruction, Analysis, and Visualization of Phylogenomic Data. Molecular biology and evolution. 2016;33(6):1635–8. 10.1093/molbev/msw046 26921390 PMC4868116

[64] Ramia M, Librado P, Casillas S, Rozas J, Barbadilla A. PopDrowser: the Population Drosophila Browser. Bioinformatics. 2012;28(4):595–6. 10.1093/bioinformatics/btr691 22180410

[65] Li H. Aligning sequence reads, clone sequences and assembly contigs with BWA-MEM. bioRxiv 13033997v2. 2013.

[66] Barnett DW, Garrison EK, Quinlan AR, Stromberg MP, Marth GT. BamTools: a C++ API and toolkit for analyzing and managing BAM files. Bioinformatics. 2011;27(12):1691–2. 10.1093/bioinformatics/btr174 21493652 PMC3106182

[67] RStudioTeam. RStudio: Integrated Development for R. RStudio, Inc., Boston, MA URL http://www.rstudio.com/. 2015.

[68] Schou MF. Fast egg collection method greatly improves randomness of egg sampling in Drosophila melanogaster. Fly (Austin). 2013;7(1):44–6. 23247611 10.4161/fly.22758 PMC3660287

[69] Voight BF, Kudaravalli S, Wen X, Pritchard JK. A map of recent positive selection in the human genome. PLoS biology. 2006;4(3):e72 16494531 10.1371/journal.pbio.0040072 PMC1382018

[70] Ferrer-Admetlla A, Liang M, Korneliussen T, Nielsen R. On detecting incomplete soft or hard selective sweeps using haplotype structure. Molecular biology and evolution. 2014;31(5):1275–91. 10.1093/molbev/msu077 24554778 PMC3995338

[71] Garud NR, Messer PW, Buzbas EO, Petrov DA. Recent selective sweeps in North American Drosophila melanogaster show signatures of soft sweeps. PLoS genetics. 2015;11(2):e1005004 10.1371/journal.pgen.1005004 25706129 PMC4338236

[72] Sabeti PC, Varilly P, Fry B, Lohmueller J, Hostetter E, Cotsapas C, et al Genome-wide detection and characterization of positive selection in human populations. Nature. 2007;449(7164):913–8. 17943131 10.1038/nature06250 PMC2687721

[73] Szpiech ZA, Hernandez RD. selscan: an efficient multithreaded program to perform EHH-based scans for positive selection. Molecular biology and evolution. 2014;31(10):2824–7. 10.1093/molbev/msu211 25015648 PMC4166924

[74] Delaneau O, Howie B, Cox AJ, Zagury JF, Marchini J. Haplotype estimation using sequencing reads. Am J Hum Genet. 2013;93(4):687–96. 10.1016/j.ajhg.2013.09.002 24094745 PMC3791270

