# Supplementary figures and images for: Multiple Independent Retroelement Insertions in the Promoter of a Stress Response Gene Have Variable Molecular and Functional Effects in Drosophila
Source: PLoS Genet. 2016 Aug 12;12(8):e1006249. doi: 10.1371/journal.pgen.1006249 (PMC4982627; doi:10.1371/journal.pgen.1006249)

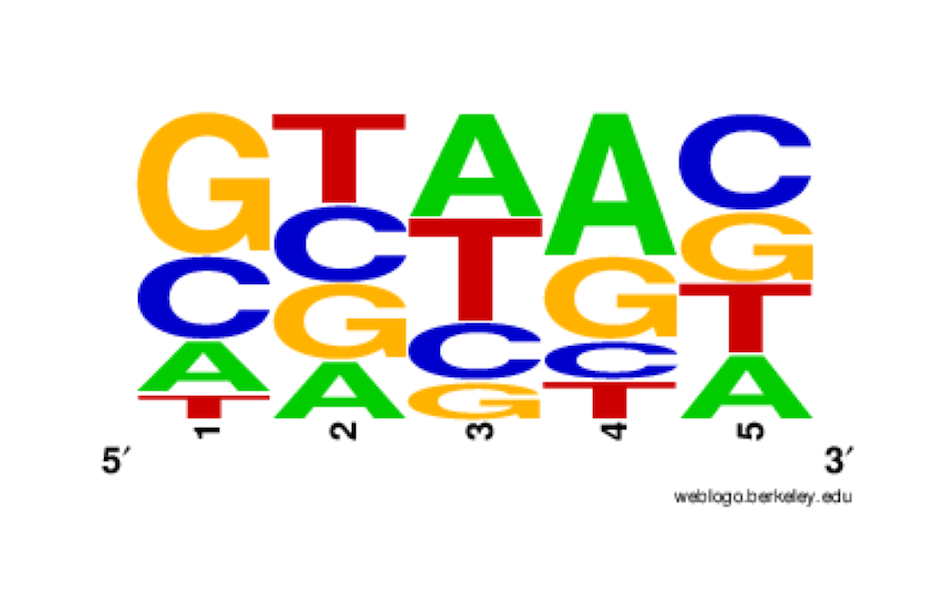

Supplement: S1 Fig — A frequency plot was built with all the TSD identified in this work, except the TSD of roo-19 and roo+7 that had four and two nucleotides instead of five, respectively, and with the 41 roo TSD motifs identified by Fiston-Lavier et al (2015) [34] (see Materials and Methods). (TIFF) [file pgen.1006249.s001.tiff]

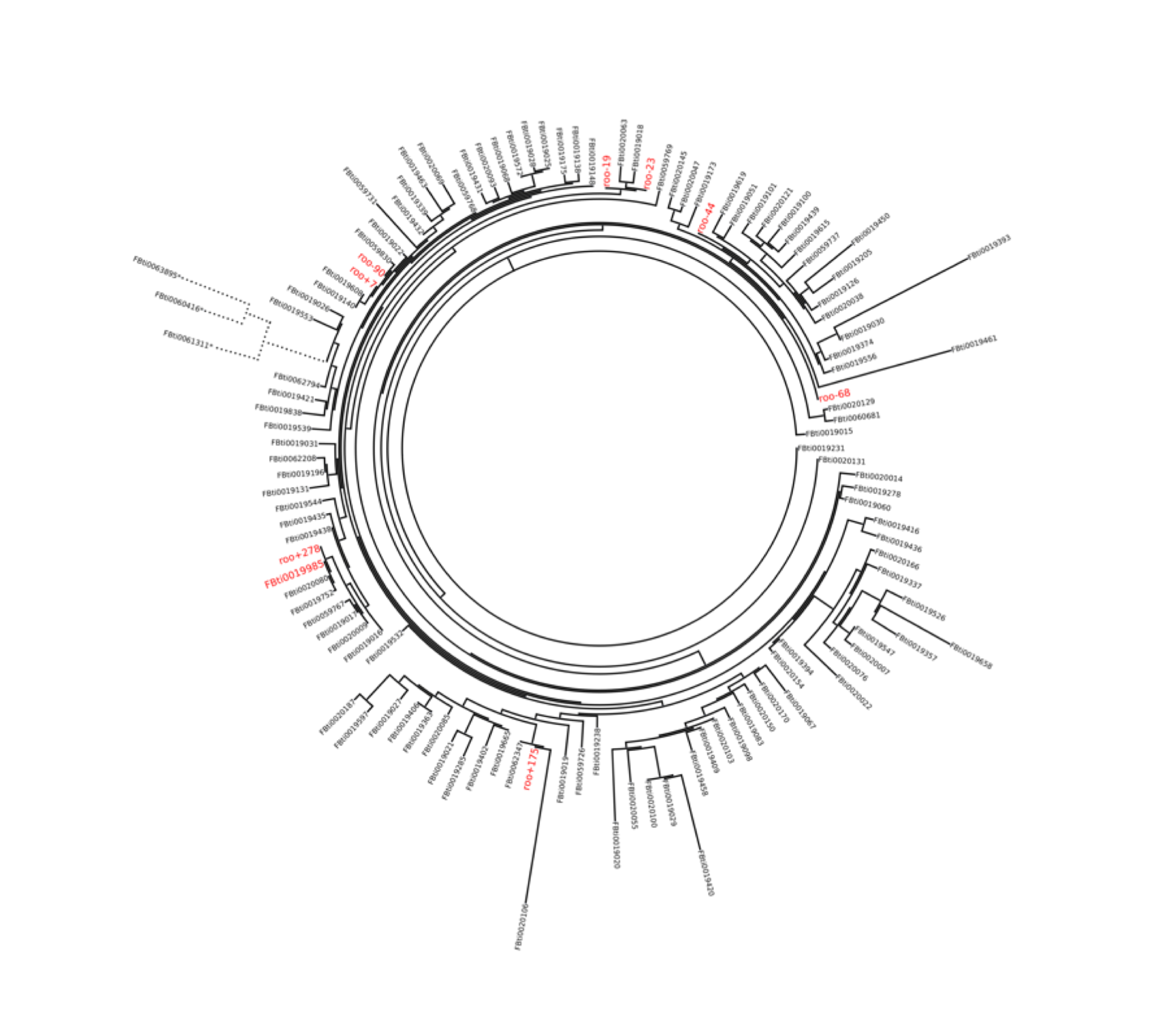

Supplement: S2 Fig — The nine roo elements sequenced in this work are depicted in red. (TIFF) [file pgen.1006249.s002.tiff]

Figure S4

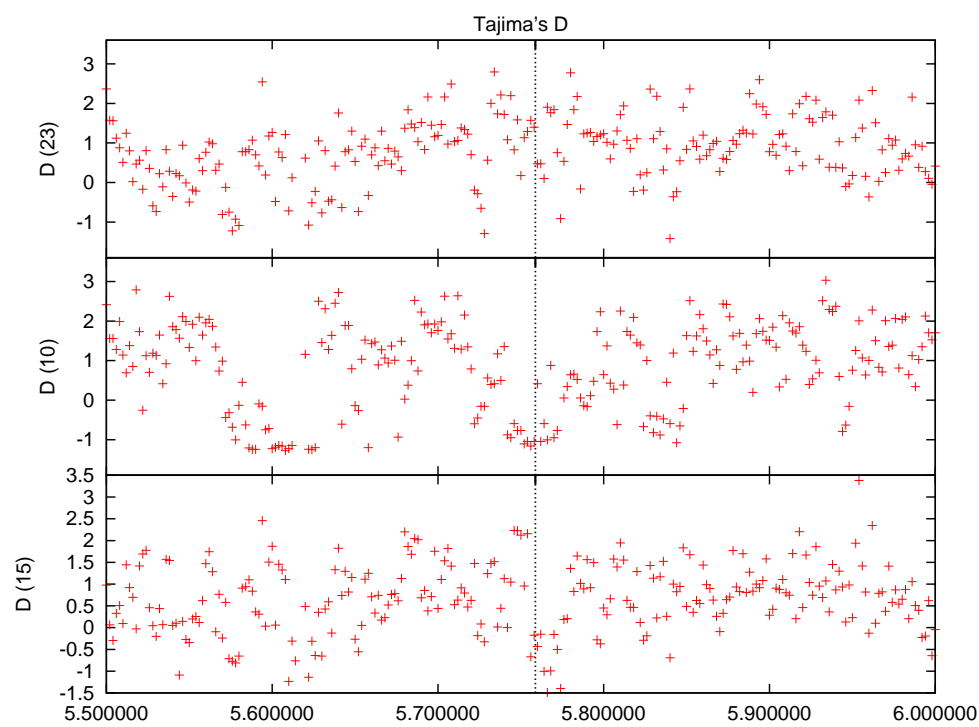

Supplement: S4 Fig — (PDF) [file pgen.1006249.s004.pdf]

Figure S5

10 strains with *FBti0019985* and 15 without

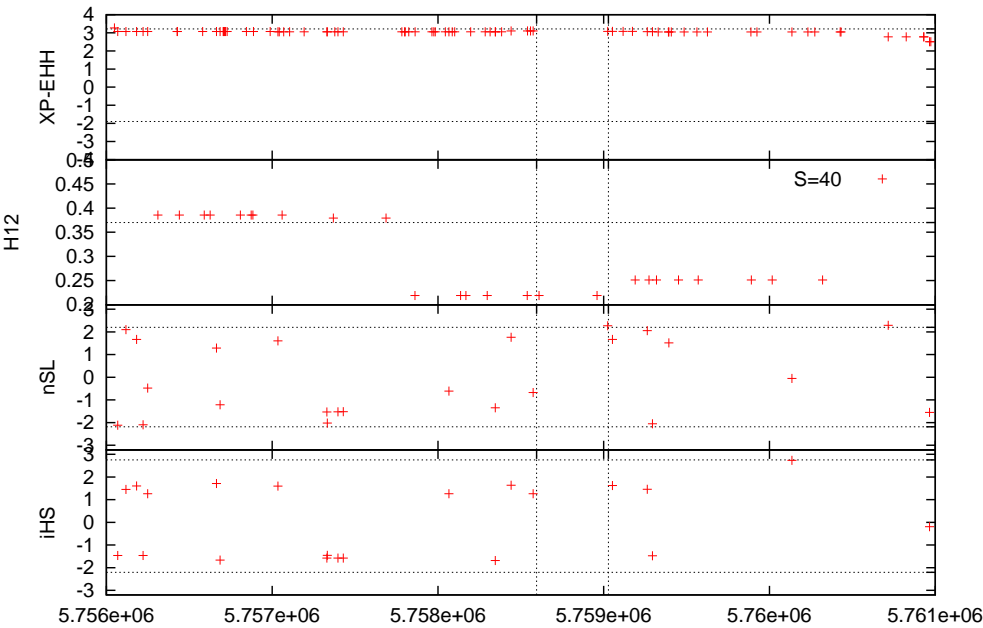

Supplement: S5 Fig — H12 was calculated on haplotypes of 40 segregating sites. All results are for the 10 strains with the FBti0019985 insertion combined with the 15 strains without any of the nine insertions, except for XP-EHH, which is calculated between the 10 strains with the FBti0019985 insertion and the 15 strains without any of the nine insertions. Horizontal dashed lines show significance levels while vertical dashed lines show the region of the insertion. (PDF) [file pgen.1006249.s005.pdf]

Figure S6

23 strains with insertion, 15 strains without insertion

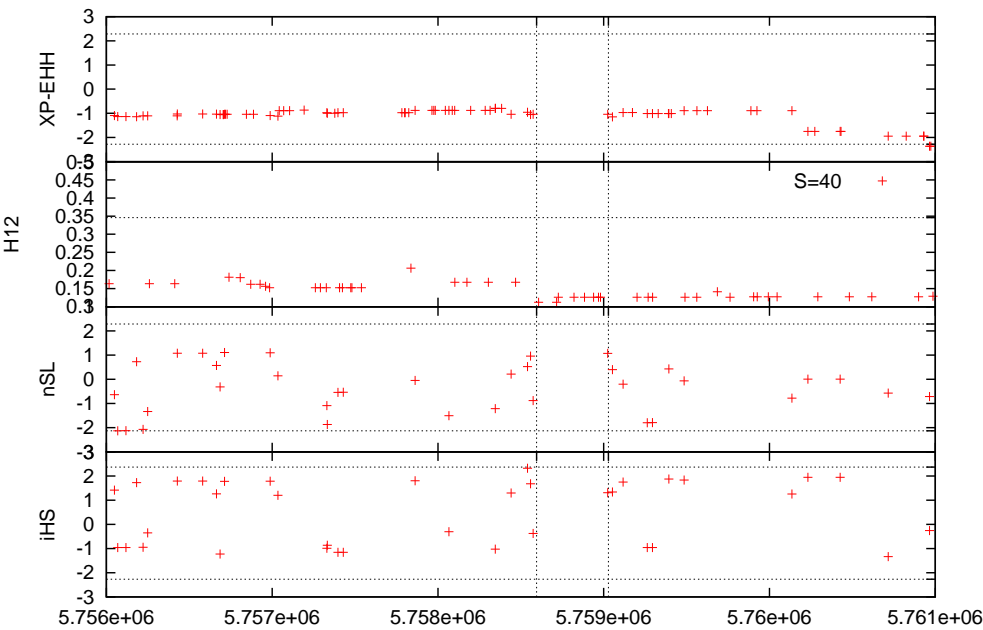

Supplement: S6 Fig — See legend of S5 Fig for details. (PDF) [file pgen.1006249.s006.pdf]
