# Supplementary material for: Multiple Independent Retroelement Insertions in the Promoter of a Stress Response Gene Have Variable Molecular and Functional Effects in Drosophila
Source: PLoS Genet. 2016 Aug 12;12(8):e1006249. doi: 10.1371/journal.pgen.1006249 (PMC4982627; doi:10.1371/journal.pgen.1006249)
Supplement: S3 Fig — Single nucleotide polymorphisms are highlighted in red. (A) Alignment of the different roo insertions analyzed in this work. For RAL-502 and RAL-857 we could only sequence a partial region of the insertion and thus we only analyzed the Inr motif. (B) Alignment of the three regions with matrix association potential. (C) Alignment of the CG18446 promoter region in the different strains analyzed. Underlined sequences are from popdrowser [64]. For additional details see Fig 3 legend. (PDF) [file pgen.1006249.s003.pdf]

**A**

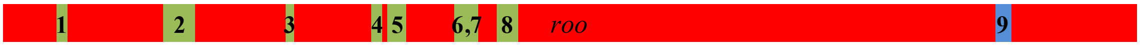


| | 1 Deaf1 | 2 Nub | 3 ara mirr caup | 4 ara mirr | 5 Bap | 6 Tin | 7 Vnd | 8 Btd | 9 INR |
|---|---|---|---|---|---|---|---|---|---|
| *FBti0019985 (RAL-639)* | TTCGTG | TATGTAAATGAA | TAACA | AAACA | TTAAGTG | CTCAAGTG | TCTCAAGTG | AGGAGGCGGG | ATCAGTT |
| *FBti0019985 (RAL-802)* | TTCGTG | TATGTAAATGAA | TAACA | AAACA | TTAAGTG | CTCAAGTG | TCTCAAGTG | AGGAGGCGGG | ATCAGTT |
| *FBti0019985 (RAL-810)* | TTCGTG | TATGTAAATGAA | TAACA | AAACA | TTAAGTG | CTCAAGTG | TCTCAAGTG | AGGAGGCGGG | ATCAGTT |
| *FBti0019985 (IV68)* | TTCGTG | TATGTAAATGAA | TAACA | AAACA | TTAAGTG | CTCAAGTG | TCTCAAGTG | **C**G**A**AGGCG**C**G | ATCAGTT |
| *roo*$_{+7}$ *(RAL- 405)* | TTCGTG | TATGTAAATGAA | TAACA | AAACA | TTAAGTG | CTCAAGTG | TCTCAAGTG | AGGAGGCGGG | ATCAGTT |
| *roo*$_{+7}$ *(RAL- 887)* | TTCGTG | TATGTAAATGAA | TAACA | AAACA | TTAAGTG | CTCAAGTG | TCTCAAGTG | AGGAGGCGGG | ATCAGTT |
| *roo*$_{+7}$ *(RAL-911)* | TTCGTG | TATGTAAATGAA | TAACA | AAACA | TTAAGTG | CTCAAGTG | TCTCAAGTG | AGGAGGCGGG | ATCAGTT |
| *roo*$_{+7}$ *(RAL-441)* | TTCGTG | TATGTAAATGAA | TAACA | AAACA | TTAAGTG | CTCAAGTG | TCTCAAGTG | AGGAGGCGGG | ATCAGTT |
| *roo*$_{+7}$ *(RAL-801)* | TTCGTG | TATGTAAATGAA | TAACA | AAACA | TTAAGTG | CTCAAGTG | TCTCAAGTG | AGGAGGCGGG | ATCAGTT |
| *roo*$_{+175}$ *(IV145)* | TTCGTG | TATGTAAATGAA | TAACA | AAACA | TTAAGTG | CTCAAGTG | TCTCAAGTG | AGGAGGCGGG | AT**T**AGTT |
| *roo*$_{+278}$ *(RAL-502)* | | | | | | | | | ATCAGTT |
| *roo*$_{-19}$ *(IV42)* | TTCGTG | TATGTAAATGAA | TAACA | AAACA | TTAAGTG | CTCAAGTG | TCTCAAGTG | AGGAGGCGGG | ATCAGTT |
| *roo*$_{-19}$ *(IV127)* | **C**TCGTG | TATGTAAATGAA | TAACA | AAACA | TTAAGTG | CTCAAGTG | TCTCAAGTG | AGGAGGCGGG | ATCAGTT |
| *roo*$_{-28}$ *(IV40)* | TTCGTG | TATGTAAATGAA | TAACA | AAACA | TTAAGTG | CTCAAGTG | TCTCAAGTG | AGGAGGCGGG | ATCAGTT |
| *roo*$_{-44}$ *(RAL-195)* | TTCGTG | TATGTAAATGAA | TAACA | AAACA | TTAAGTG | CTCAAGTG | TCTCAAGTG | AGGAGGCGGG | ATCAGTT |
| *roo*$_{-44}$ *(RAL-383)* | TTCGTG | TATGTAAATGAA | TAACA | AAACA | TTAAGTG | CTCAAGTG | TCTCAAGTG | AGGAGGCGGG | ATCAGTT |
| *roo*$_{-68}$ *(RAL-75)* | TTCGTG | TATGTAAATGAA | TAACA | AAACA | TTAAGTG | CTCAAGTG | TCTCAAGTG | AGGAGGCGGG | A**C**CAGTT |
| *roo*$_{-68}$ *(RAL-716)* | TTCGTG | TATGTAAATGAA | TAACA | AAACA | TTAAGTG | CTCAAGTG | TCTCAAGTG | AGGAGGCGGG | ATCAGTT |
| *roo*$_{-68}$ *(IV69)* | TTCGTG | TATGTAAATGAA | TAACA | AAACA | TTAAGTG | CTCAAGTG | TCTCAAGTG | AGGAGGCGGG | ATCAGTT |
| *roo*$_{-90}$ *(RAL-21)* | TTCGTG | TATGTAAATGAA | TAACA | AAACA | TTAAGTG | CTCAAGTG | TCTCAAGTG | AGGAGGCGGG | ATCAGTT |
| *roo*$_{-90}$ *(RAL-88)* | TTCGTG | TATGTAAATGAA | TAACA | AAACA | TTAAGTG | CTCAAGTG | TCTCAAGTG | AGGAGGCGGG | ATCAGTT |
| *roo*$_{-90}$ *(RAL-177)* | TTCGTG | TATGTAAATGAA | TAACA | AAACA | TTAAGTG | CTCAAGTG | TCTCAAGTG | AGGAGGCGGG | ATCAGTT |
| *roo*$_{-90}$ *(RAL-737)* | TT**GCAT** | TATGTAAATGAA | TAACA | AAACA | TTAAGTG | CTCAAGTG | TCTCAAGTG | AGGAGGCGGG | ATCAGTT |
| *roo*$_{-90}$ *(RAL-820)* | TTCGTG | TATGTAAATGAA | TAACA | AAACA | TTAAGTG | CTCAAGTG | TCTCAAGTG | AGGAGGCGGG | ATCAGTT |
| *roo*$_{-90}$ *(RAL-857)* | | | | | | | | | ATCAGTT |
| *roo*$_{-90}$ *(IV50)* | TTCGTG | TATGTAAATGAA | TAACA | AAACA | TTAAGTG | CTCAAGTG | TCTCAAGTG | AGGAGGCGGG | ATCAGTT |
| | * | ************ | ***** | ***** | ******* | ******** | ********* | * ***** * | * **** |

**B**

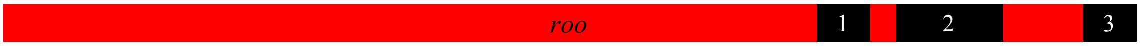


| | 1 | 2 | 3 |
|---|---|---|---|
| *FBti0019985 (RAL-639)* | GTAGGCCATTTACTTTAAGA | ATGTCACCTATTTAAACCGAAGATATTTCCAAATAAA ATCA | TTCTTATTTGGGATTTTACA |
| *FBti0019985 (RAL-802)* | GTAGGCCATTTACTTTAAGA | ATGTCACCTATTTAAACCGAAGATATTTCCAAATAAA ATCA | TTCTTATTTGGGATTTTACA |
| *FBti0019985 (RAL-810)* | GTAGGCCATTTACTTTAAGA | ATGTCACCTATTTAAACCGAAGATATTTCCAAATAAA ATCA | TTCTTATTTGGGATTTTACA |
| *FBti0019985 (IV68)* | GTAGGCCATTTACTTTAAGA | ATGTCACCTATTTAAACCGAAGATATTTCCAAATAAA ATCA | TTCTTATTTGGGATTTTACA |
| *roo*$_{+7}$ *(RAL- 405)* | GTAGGCCATTTACTTTAAGA | ATGTCACCTATTTAAACCGAAGATATTTCCAAATAAA ATCA | TTCTTATTTGGGATTTTACA |
| *roo*$_{+7}$ *(RAL- 887)* | GTAGGCCATTTACTTTAAGA | ATGTCACCTATTTAAACCGAAGATATTTCCAAATAAA ATCA | TTCTTATTTGGGATTTTACA |
| *roo*$_{+7}$ *(RAL-911)* | GTAGGCCATTTACTTTAAGA | ATGTCACCTATTTAAACCGAAGATATTTCCAAATAAA ATCA | TTCTTATTTGGGATTTTACA |
| *roo*$_{+7}$ *(RAL-441)* | GTAGGCCATTTACTTTAAGA | ATGTCACCTATTTAAACCGAAGATATTTCCAAATAAA ATCA | TTCTTATTTGGGATTTTACA |
| *roo*$_{+7}$ *(RAL-801)* | GTAGGCCATTTACTTTAAGA | ATGTCACCTATTTAAACCGAAGATATTTCCAAATAAA ATCA | TTCTTATTTGGGATTTTACA |
| *roo*$_{+175}$ *(IV145)* | GTAGGCCATTTACTTTAAGA | ATGTCACCTATTTAAACCGAAGATATTTCCAAATAAA ATTA | TTCTCATTTGGGATTTTACA |
| *roo*$_{+278}$ *(RAL-502)* | GTAGGCCATTTACTTTAAGA | ATGTCACCTATTTAAACCGAAGATATTTCCAAATAAA ATCA | TTCTTATTTGGGATTTTACA |
| *roo*$_{-19}$ *(IV42)* | GTAGGCCATTTACTTTAAGA | ATGTCACCTATTTAAACCGAAGATATTTCCAAATAAA ATCA | TTCTTATTTGGGATTTTACA |
| *roo*$_{-19}$ *(IV127)* | GTAGGCCATTTACTTTAAGA | ATGTCACCTATTTAAACCGAAGATATTTCCAAATAAA ATCA | TTCTTATTTGGGATTTTACA |
| *roo*$_{-28}$ *(IV40)* | GTAGGCCATTTACTTTAAGA | ATGTCACCTATTTAAACCGAAGATATTTCCAAATAAA ATCA | TTCTTATTTGGGATTTTACA |
| *roo*$_{-44}$ *(RAL-195)* | GTAGGCCATTTACTTTAAGA | ATGTCACCTATTTAAACCGAAGATATTTCCAAATAAA ATCA | TTCTTATTTGGGATTTTACA |
| *roo*$_{-44}$ *(RAL-383)* | GTAGGCCATTTACTTTAAGA | ATGTCACCTATTTAAACCGAAGATATTTCCAAATAAA ATCA | TTCTTATTTGGGATTTTACA |
| *roo*$_{-68}$ *(RAL-75)* | GTAGGCCATTTACTTTAAGA | ATGTCACCTATTTAAACCGAAGATATTTCCAAATAAA ACCA | TTCTCATTTGGGATTTTACA |
| *roo*$_{-68}$ *(RAL-716)* | GTAGGCCATTTACTTTAAGA | ATGTCACCTATTTAAACCGAAGATATTTCCAAATAAA ATCA | TTCTTATTTGGGATTTTACA |
| *roo*$_{-68}$ *(IV69)* | GTAGGCCATTTACTTTAAGA | ATGTCACCTATTTAAACCGAAGATATTTCCAAATAAA ATCA | TTCTTATTTGGGATTTTACA |
| *roo*$_{-90}$ *(RAL-21)* | GTAGGCCATTTACTTTAAGA | ATGTCACCTATTTAAACCGAAGATATTTCCAAATAAA ATCA | TTCTTATTTGGGATTTTACA |
| *roo*$_{-90}$ *(RAL-88)* | GTAGGTCATTTACTTTAAGA | ATGTCACCTATTTAAACCGAAGATATTTCCAAATAAA ATCA | TTCTTATTTGGGATTTTACA |
| *roo*$_{-90}$ *(RAL-177)* | GTATGCCATTTACTTTAAGA | ATGTCACCTATTTAAACCGAAGATATTTCCAAATAAA ATCA | TTCTTATTTGGGATTTCACA |
| *roo*$_{-90}$ *(RAL-737)* | GTAGGCCATTTACTTTAAGA | ATGTCACCTATTTAAACCGAAGATATTTCCAAATAAA ATCA | TTCTTATTTGGGATTTTACA |
| *roo*$_{-90}$ *(RAL-820)* | GTAGGCCATTTACTTTAAGA | ATGTCACCTATTTAAACCGAAGATATTTCCAAATAAA ATCA | TTCTTATTTGGGATTTTACA |
| *roo*$_{-90}$ *(RAL-857)* | A- - GGCCATTTACTTTAAGA | ATGTCACCTATTTAAACCGAAGATATTTCCAAATAAA ATCA | TTCTTATTTGGGATTTTACA |
| *roo*$_{-90}$ *(IV50)* | GTAGGCCATTTACTTTAAGA | ATGTCACCTATTTAAACCGAAGATATTTCCAAATAAA ATCA | TTCTTATTTGGGATTTTACA |
| | * ** * ** * ** * ** ** * * | * *** ** ************** * *************** * * | **** *** *** **** *** |

**C**

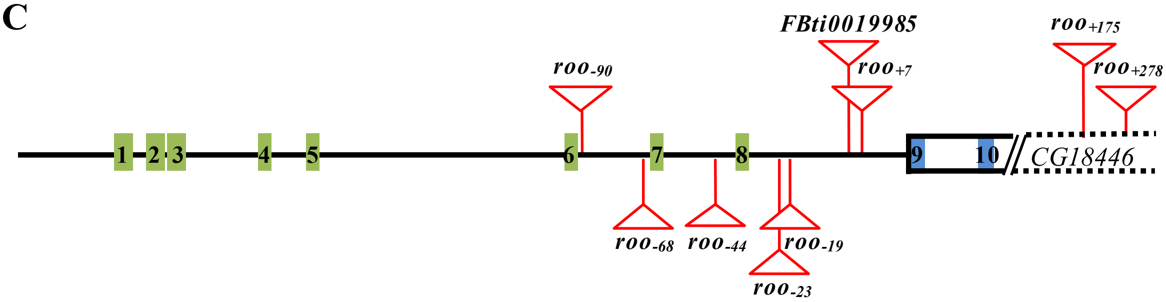


| | 1 Abd-B | 2 ct | 3 eve zen | 4 Optix | 5 Optix | 6 ara mirr | 7 ara mirr caup | 8 ara mirr | 9 INR | 10 DPE |
|---|---|---|---|---|---|---|---|---|---|---|
| FBti0019985 (RAL-639) | TTTATGA | TTGAAC | CTAATGA | TGATA | TGATA | AAACA | TAACA | AAACA | TCAGTC | A**T**TTGT |
| FBti0019985 (RAL-802) | TTTTATG | TTGAAC | CTAATGA | TGATA | TGATA | AAACA | TAACA | AAACA | T**T**AGTC | AGTTGT |
| FBti0019985 (RAL-810) | TTTATGA | TTGAAC | CTAATGA | TGATA | TGATA | AAACA | TAACA | AAACA | T**T**AGTC | AGTTGT |
| FBti0019985 (IV68) | ----------- | ----------- | ----------- | -------- | -------- | -------- | ------- | -------- | T**T**AGTC | AGTTGT |
| roo$_{+7}$ (RAL- 405) | TTTATGA | TTGAAC | CTAATGA | TGATA | TGATA | AAACA | TAACA | AAACA | TCAGTC | AGTTGT |
| roo$_{+7}$ (RAL- 887) | TTTATGA | TTGAAC | CTAATGA | TGATA | TGATA | AAACA | TAACA | AAACA | TCAGTC | AGTTGT |
| roo$_{+7}$ (RAL-911) | TTTATGA | TTGAAC | CTAATGA | TGATA | TGATA | AAACA | TAACA | AAACA | TCAGTC | AGTTGT |
| roo$_{+7}$ (RAL-441) | TTTATGA | TTGAAC | CTAATGA | TGATA | TGATA | AA**CCC** | TAACA | AAACA | TCAGTC | AGTTGT |
| roo$_{+7}$ (RAL-801) | TTTATGA | TTGAAC | CTAATGA | TGATA | TGATA | AAACA | TAACA | AAACA | TCAGTC | AGTTGT |
| roo$_{+175}$ (IV145) | TTTATGA | TTGAAC | CTAATGA | TGATA | TGATA | AAACA | TAACA | AAACA | TCAGTC | AGTTGT |
| roo$_{-19}$ (IV42) | TTTATGA | TTGAAC | CTAATGA | TGATA | TGATA | AAACA | TAACA | AAACA | T**T**AGTC | AGTTGT |
| roo$_{-19}$ (IV127) | ----------- | ----------- | ----------- | -------- | -------- | AAACA | TAACA | AAACA | T**T**AGTC | AGTTGT |
| roo$_{-28}$ (IV40) | ----------- | ----------- | ----------- | TGATA | TGATA | AAACA | TAACA | AAACA | TCAGTC | AGTTGT |
| roo$_{-44}$ (RAL-195) | TTT**TATG** | TTGAAC | CTAATGA | TGATA | TGATA | AAACA | TAACA | AAACA | T**T**AGTC | AGTTGT |
| roo$_{-44}$ (RAL-383) | TTTATGA | TTGAAC | CTAATGA | TGATA | TGATA | AAACA | TAACA | AAACA | T**T**AGTC | AGTTGT |
| roo$_{-68}$ (RAL-75) | TTTATGA | TTGAAC | CTAATGA | TGATA | TGATA | AAACA | TAACA | AAACA | TCAGTC | AGTTGT |
| roo$_{-68}$ (RAL-716) | TTTATGA | TTGAAC | CTAATGA | TGATA | TGATA | AAACA | TAACA | AAACA | T**T**AGTC | AGTTGT |
| roo$_{-68}$ (IV69) | TTTATGA | TTGAAC | CTAATGA | TGATA | TGATA | AAACA | TAACA | AAACA | TCAGTC | AGTTGT |
| roo$_{-90}$ (RAL-21) | TTTATGA | TTGAAC | CTAATGA | TGATA | TGATA | AAACA | TAACA | AAACA | TCAGTC | AGTTGT |
| roo$_{-90}$ (RAL-88) | TTTATGA | TTGAAC | CTAATGA | TGATA | TGATA | AAACA | TAACA | AAACA | T**T**AGTC | AGTTGT |
| roo$_{-90}$ (RAL-177) | TTTATGA | TTGAAC | CTAATGA | TGATA | TGATA | AAACA | TAACA | AA**TT**A | T**T**AGTC | AGTTGT |
| roo$_{-90}$ (RAL-737) | TTTATGA | TTGAAC | CTAATGA | TGATA | TGATA | AAACA | TAACA | AAACA | T**T**AGTC | A**T**TTGT |
| roo$_{-90}$ (RAL-820) | TTTATGA | TTGAAC | CTAATGA | TGATA | TGATA | AAACA | TAACA | AAACA | T**T**AGTC | AGTTGT |
| roo$_{-90}$ (RAL-857) | TTTATGA | TTGAAC | CTAATGA | TGATA | TGATA | AAACA | TAACA | AAACA | TCAGTC | AGTTGT |
| roo$_{-90}$ (IV50) | TTTATGA | TTGAAC | CTAATGA | TGATA | TGATA | AAACA | TAACA | AAACA | TCAGTC | AGTTGT |
| roo- (RAL-371) | TTTATGA | TTGAAC | CTAATGA | TGATA | TGATA | AAACA | TAACA | AAACA | TCAGTC | AGTTGT |
| roo- (RAL-391) | TTTATGA | TTGAAC | CTAATGA | TGATA | TGATA | AAACA | TAACA | AAACA | TCAGTC | AGTTGT |
| roo- (RAL-783) | TTTATGA | TTGAAC | CTAATGA | TGATA | TGATA | AAACA | TAACA | AAACA | TCAGTC | AGTTGT |
| roo- (RAL-908) | TTTATGA | TTGAAC | CTAATGA | TGATA | TGATA | AAACA | TAACA | AAACA | TCAGTC | AGTTGT |
| roo- (IV22) | TTTATGA | TTGAAC | CTAATGA | TGATA | TGATA | AAACA | TAACA | AAACA | TCAGTC | AGTTGT |
| | * * * | * * * * * * | * * * * * * * | * * * * * | * * * * * | * * * | * * * * * | * * * | * * * * * | * * * * * |
