# Supplementary material for: Multiple Independent Retroelement Insertions in the Promoter of a Stress Response Gene Have Variable Molecular and Functional Effects in Drosophila
Source: PLoS Genet. 2016 Aug 12;12(8):e1006249. doi: 10.1371/journal.pgen.1006249 (PMC4982627; doi:10.1371/journal.pgen.1006249)
Supplement: S2 Table — (A) Transcription factor binding sites and promoter motifs, and (B) Matrix Associated regions, found in FBti0019985. (C) Transcription factor binding sites and promoter motifs found in the CG18446 promoter region. (DOCX) [file pgen.1006249.s008.docx]

**Table S2A. Transcription factor binding sites and promoter motifs found inside *FBti0019985.***

| **Model name** | **Relative score** | **Chromosome** | **Start** | **End** | **Strand** | **Predicted site sequence** |
| --- | --- | --- | --- | --- | --- | --- |
| Deaf1 | 1.000 | 2R | 9871110 | 9871115 | -1 | ttcgtg |
| mirr | 0.996 | 2R | 9871197 | 9871201 | -1 | taaca |
| ara | 1.000 | 2R | 9871197 | 9871201 | -1 | taaca |
| caup | 1.000 | 2R | 9871197 | 9871201 | -1 | taaca |
| ara | 0.995 | 2R | 9871230 | 9871234 | 1 | aaaca |
| mirr | 0.999 | 2R | 9871230 | 9871234 | 1 | aaaca |
| bap | 0.999 | 2R | 9871241 | 9871247 | 1 | ttaagtg |
| vnd | 0.995 | 2R | 9871266 | 9871274 | -1 | tctcaagtg |
| Nub | NA^(a)^ | 2R | 9871156 | 9871167 | 1 | tatgtaaatgaa |
| Tin | NA^(a)^ | 2R | 9871266 | 9871273 | -1 | ctcaagtg |
| Btd | NA^(a)^ | 2R | 9871281 | 9871290 | -1 | aggaggcggg |
| INR | NA^(b)^ | 2R | 9871470 | 9871476 | 1 | atcagtt |

**Table S2B. Genomic regions with matrix association potential (MARs) found inside *FBti0019985*.**

| **Model name** | **Chromosome** | **Start** | **End** | **Strand** | **Predicted site sequence** |
| --- | --- | --- | --- | --- | --- |
| MAR | 2R | 9871403 | 9871422 | 1 | gtaggccatttactttaaga |
| MAR | 2R | 9871433 | 9871473 | 1 | atgtcacctatttaaaccgaagatatttccaaataaaatca |
| MAR | 2R | 9871504 | 9871523 | 1 | ttcttatttgggattttaca |

**Table S2C. Transcription factor binding sites and promoter motifs found in *CG18446* promoter region.**

| **Model name** | **Relative score** | **Chromosome** | **Start** | **End** | **Strand** | **Predicted site sequence** |
| --- | --- | --- | --- | --- | --- | --- |
| Abd-B | 1.000 | 2R | 9870850 | 9870856 | -1 | tttatga |
| ct | 0.999 | 2R | 9870861 | 9870866 | 1 | ttgaac |
| eve | 0.999 | 2R | 9870868 | 9870874 | 1 | ctaatga |
| zen | 1.000 | 2R | 9870868 | 9870874 | 1 | ctaatga |
| Optix | 1.000 | 2R | 9870898 | 9870902 | -1 | tgata |
| Optix | 1.000 | 2R | 9870914 | 9870918 | -1 | tgata |
| ara | 0.995 | 2R | 9871999 | 9871003 | -1 | aaaca |
| mirr | 0.999 | 2R | 9871999 | 9871003 | -1 | aaaca |
| mirr | 0.996 | 2R | 9871028 | 9871032 | -1 | taaca |
| ara | 1.000 | 2R | 9871028 | 9871032 | -1 | taaca |
| caup | 1.000 | 2R | 9871028 | 9871032 | -1 | taaca |
| ara | 0.995 | 2R | 9871056 | 9871060 | 1 | aaaca |
| mirr | 0.999 | 2R | 9871056 | 9871060 | 1 | aaaca |
| INR | NA^(b)^ | 2R | 9871547 | 9871552 | 1 | tcagtc |
| DPE | NA^(b)^ | 2R | 9871576 | 9871581 | 1 | agttgt |

^(a)^TFBS described in Batut et al 2013. These TFBS are not included in the JASPAR database.

^(b)^Core promoter motifs described in Juven-Gershon and Kadonaga 2010. These TFBS are not included in the JASPAR database.
