# Supplementary material for: Multiple Independent Retroelement Insertions in the Promoter of a Stress Response Gene Have Variable Molecular and Functional Effects in Drosophila
Source: PLoS Genet. 2016 Aug 12;12(8):e1006249. doi: 10.1371/journal.pgen.1006249 (PMC4982627; doi:10.1371/journal.pgen.1006249)
Supplement: S4 Table — (DOCX) [file pgen.1006249.s010.docx]

Table S4. Results of the different statistics used to infer positive selection in the region flanking the nine solo-LTR insertions.

| **Region analyzed** | **10 strains with *FBti0019985* insertion** | **15 strains without insertion** | **23 strains with one of the nine *roo* insertions** |
| --- | --- | --- | --- |
| *S* in 2R: 5,758,000-5,760,000 | 5 | 12 | 10 |
| S/L in 2R:5,758,000-5,760,000 | **0.0025** | 0.006 | 0.0050 |
| S/L in 2R | 0.0149 | 0.0211 | 0.0240 |
| S/L in whole genome | 0.0135 | 0.0185 | 0.0206 |
| Tajima's D | **-1.0446** | -0.1724 | 1.3970 |
| iHS | **3.3470** | NA | **2.3200** |
| nSL | 2.2730 | NA | -1.7970 |
| H_12_ | **0.16** | NA | 0.023 |
